# Supplementary material for: Targeting TAZ-TEAD in minimal residual disease enhances the duration of targeted therapy in melanoma models
Source: Nat Commun. 2025 Nov 5;16:9655. doi: 10.1038/s41467-025-64682-7 (PMC12589586; doi:10.1038/s41467-025-64682-7)
Supplement: Supplementary file 1 — Supplementary Information [file 41467_2025_64682_MOESM1_ESM.pdf]

Supplementary Information

Supplementary Figure Legends to: Targeting TAZ-TEAD in minimal residual disease enhances the duration of targeted therapy in melanoma models

Supplementary Figure 1: The melanoma invasive phenotype is associated with increased YAP1/TAZ-TEAD activity. A Box plots of MITF, NGFR, KDM5B, and Melan A expression levels by cell state in a scRNA-seq dataset of patient-derived xenograft melanomas following BRAFi + MEKi from Rambow et al. [1]. The FindMarkers() function in Seurat was used to determine differentially expressed genes between groups of cells. Cells from one group were compared against all other cells. Statistical values for genes expressed in at least 10% of cells in a group and with a minimum of 0.25 log2 fold change differences were reported. P-adjusted values were calculated using the BHFDR method for all reported genes. Box plots are made with ggplot2::geom_boxplot() using default summary statistic parameters, which show the 25% quantile, median, and 75% quantile for the lower bound, center line, and upper bound of the box, respectively. Box plot whiskers are drawn to the lowest or highest data point within 1.5 * IQR from the lower or upper bound of the box for the lower and upper whiskers, respectively. **B** A375 parental, A375 2.18 SOX10 KO, A375 4.21 SOX10 KO cells and MeWo parental, MeWo 2.1 SOX10 KO, and MeWo 4.11 SOX10 KO cell lysates were analyzed by Western blotting with the indicated antibodies. The experiment was repeated independently three times with similar results. **C** Enrichment plots of Harvey Melanoma Up gene signature [2] for MeWo and A375 CRISPR SOX10 KO cells vs parental cells. ***p<0.001 BHFDR. **D** A375 parental, A375 2.18 SOX10 KO, A375 4.21 SOX10 KO cells and MeWo parental, MeWo 2.1 SOX10 KO, and MeWo 4.11 SOX10 KO cell lysates were analyzed by Western blotting with the antibodies indicated. The experiment was repeated independently three times with similar results. **E** A375 parental, A375 2.18 SOX10 KO, A375 4.21 SOX10 KO cells and MeWo parental, MeWo 2.1 SOX10 KO, and MeWo 4.11

SOX10 KO cell lysates were analyzed by Western blotting with the antibodies indicated. The experiment was repeated independently three times with similar results.

Ott et al., Supplementary Figure 1

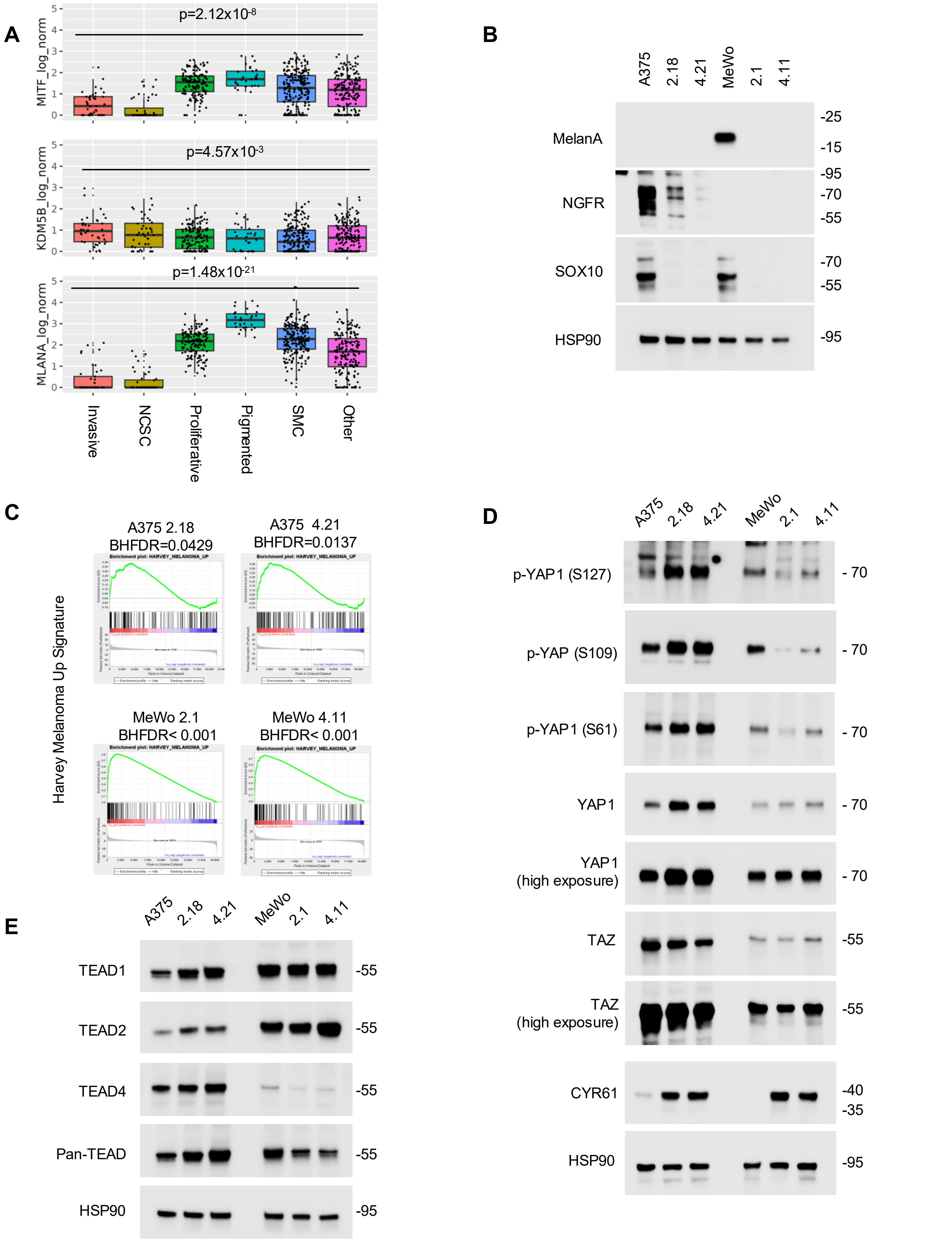

Supplementary Figure 2: c-Jun expression is enhanced in SOX10 KO cells contributes to regulation of CTGF and CYR61 expression. A Enrichment plots of predicted AP-1 targets in invasive melanoma cell lines gene signature [3] for MeWo and A375 CRISPR SOX10 KO cells vs parental cells. **B** Scatter plot showing negative log10-transformed BHFDR values from binary motif enrichment analysis statistical results for significantly up-regulated versus not-significant peaks when comparing ATAC-seq data of SOX10-negative (n=3) to SOX10-positive (n=6) cell lines. **C** UCSC genome track plots of CYR61 (top) and CTGF (bottom), showing genomic coordinates, ATAC-seq data for nine melanoma cell lines, gene intron and exon regions, and predicted binding motifs. For the melanoma ATAC-seq data, the top six rows are SOX10-positive while the bottom three rows are SOX10-negative. Dark grey and black regions represent increased open chromatin. The UCSC track hub data (http://ucsctracks.aertslab.org/papers/wouters_human_melanoma/hub.txt) are from Wouters et al. **D** Barplot of RNA-seq data showing c-Jun normalized read count values in A375 parental, MeWo parental, and their respective SOX10 KO cell lines. *$p<0.05$, **$p<0.01$, One-way ANOVA. **E** A375 parental, A375 2.18 SOX10 KO, A375 4.21 SOX10 KO cell lysates and MeWo parental, MeWo 2.1 SOX10 KO, and MeWo 4.11 SOX10 KO cell lysates were analyzed by Western blotting with the antibodies indicated. The experiment was repeated independently three times with similar results. **F** A375 SOX10 KO cells were untreated or treated with reagent alone, non-targeting control siRNA, or si c-Jun. After 72 hrs, cells were lysed and lysates were analyzed by Western blotting with the antibodies indicated.

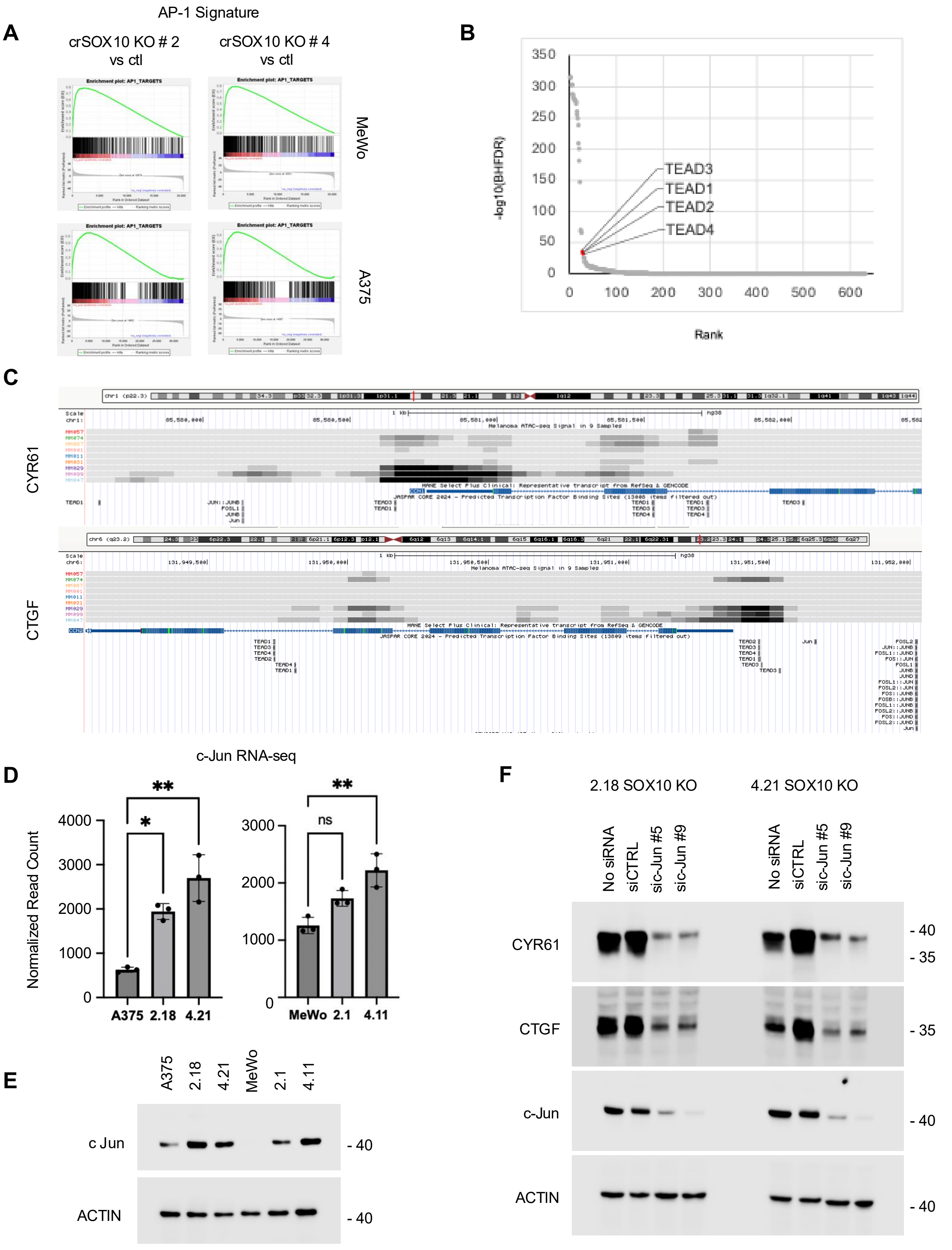
AP-1 Signature
A
crSOX10 KO # 2
vs ctl
crSOX10 KO # 4
vs ctl
Enrichment plot: AP1_TARGETS
MeWo
A375
B
-log10(BHFDR)
TEAD3
TEAD1
TEAD2
TEAD4
Rank
C
CYR61
CTGF
Melanoma ATAC-seq Signal in 9 Samples
MANE Select Plus Clinical: Representative transcript from RefSeq & GENCODE
JASPAR CORE 2024 - Predicted Transcription Factor Binding Sites (13889 items filtered out)
D
c-Jun RNA-seq
Normalized Read Count
A375
2.18
4.21
MeWo
2.1
4.11
ns
E
c Jun
ACTIN
- 40
F
2.18 SOX10 KO
4.21 SOX10 KO
No siRNA
siCTRL
sic-Jun #5
sic-Jun #9
CYR61
CTGF
c-Jun
ACTIN
- 40
- 35

Supplementary Figure 3: Depletion of TAZ and transcriptomic effects. A Heatmap showing GSEA NES for the hallmark gene sets in SOX10 KO cells following knockdown of either YAP1 or TAZ compared to non-targeting siRNA-treated cells. NES values are displayed for enriched gene sets using a BHFDR cutoff of 0.05. Shown is the mean from three independent replicates generated for each cell line. **B** Bar plot of five highest and lowest scoring Hallmark signatures following knockdown of TAZ or YAP1 in 2.18 and 4.21 SOX10 KO cell lines. Signatures were selected based on which signatures had the highest magnitude in either direction of the average Normalized Enrichment Score (NES) across all Gene Set Enrichment Analysis (GSEA) samples. **C** Density plot of the absolute values of Normalized Enrichment Scores (NES) in the Hallmark gene signature collection across siTAZ and siYAP lines. Vertical lines were added to indicate the median values for each curve.

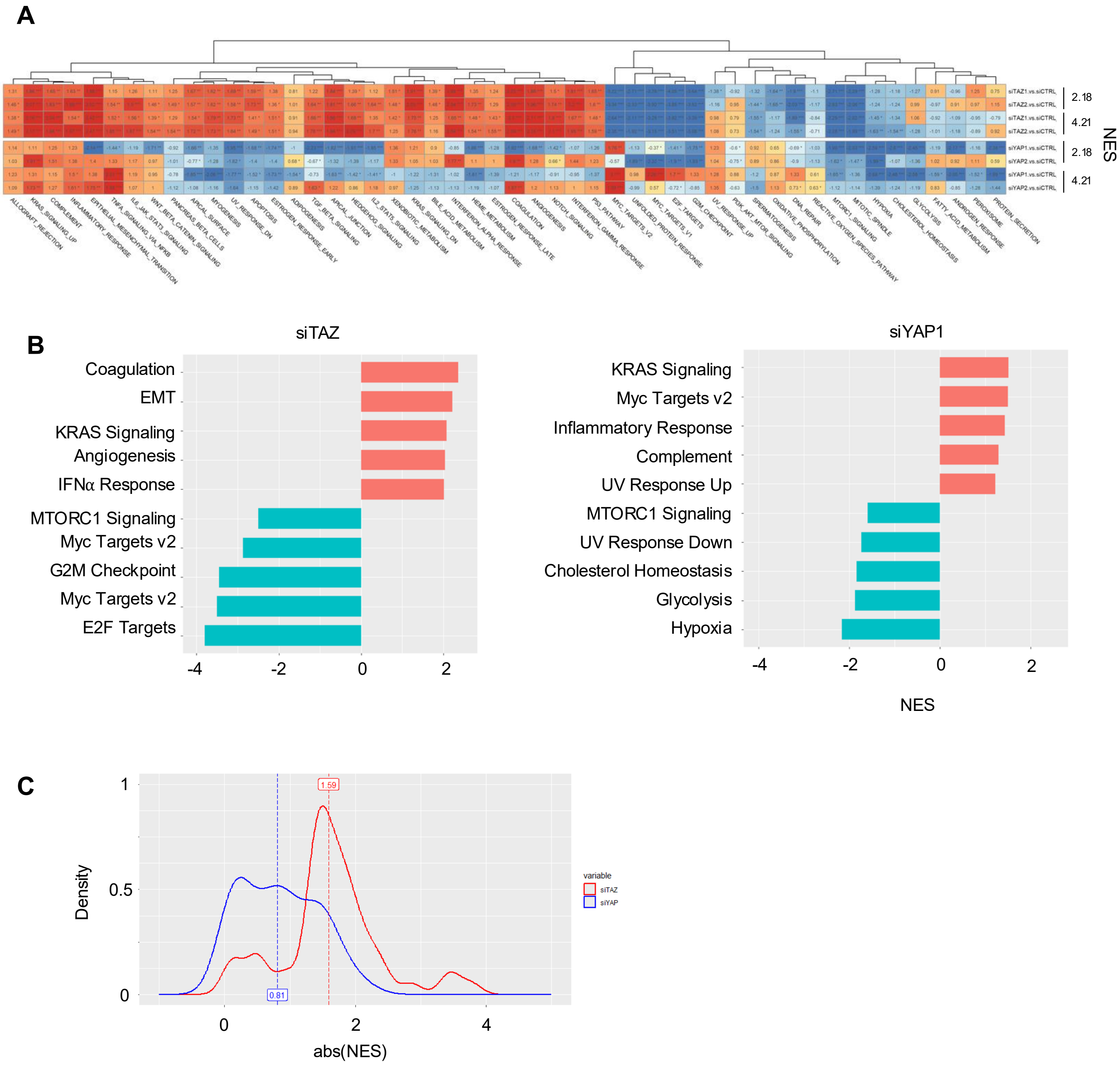
A
siTAZ1.vs.siCTRL
siTAZ2.vs.siCTRL
siYAP1.vs.siCTRL
siYAP2.vs.siCTRL
2.18
4.21
NES
ALLOGRAFT_REJECTION
KRAS_SIGNALING_UP
COMPLEMENT
INFLAMMATORY_RESPONSE
EPITHELIAL_MESENCHYMAL_TRANSITION
TNFA_SIGNALING_VIA_NFKB
IL6_JAK_STAT3_SIGNALING
WNT_BETA_CATENIN_SIGNALING
PANCREAS_BETA_CELLS
APICAL_SURFACE
MYOGENESIS
UV_RESPONSE_DN
APOPTOSIS
ESTROGEN_RESPONSE_EARLY
ADIPOGENESIS
TGF_BETA_SIGNALING
APICAL_JUNCTION
HEDGEHOG_SIGNALING
IL2_STAT5_SIGNALING
XENOBIOTIC_METABOLISM
KRAS_SIGNALING_DN
BILE_ACID_METABOLISM
INTERFERON_ALPHA_RESPONSE
HEME_METABOLISM
ESTROGEN_RESPONSE_LATE
COAGULATION
ANGIOGENESIS
NOTCH_SIGNALING
INTERFERON_GAMMA_RESPONSE
P53_PATHWAY
MYC_TARGETS_V2
UNFOLDED_PROTEIN_RESPONSE
MYC_TARGETS_V1
E2F_TARGETS
G2M_CHECKPOINT
UV_RESPONSE_UP
PI3K_AKT_MTOR_SIGNALING
SPERMATOGENESIS
OXIDATIVE_PHOSPHORYLATION
DNA_REPAIR
REACTIVE_OXYGEN_SPECIES_PATHWAY
MTORC1_SIGNALING
MITOTIC_SPINDLE
HYPOXIA
CHOLESTEROL_HOMEOSTASIS
GLYCOLYSIS
FATTY_ACID_METABOLISM
ANDROGEN_RESPONSE
PEROXISOME
PROTEIN_SECRETION
B
siTAZ
Coagulation
EMT
KRAS Signaling
Angiogenesis
IFNα Response
MTORC1 Signaling
Myc Targets v2
G2M Checkpoint
Myc Targets v2
E2F Targets
-4
-2
0
2
siYAP1
KRAS Signaling
Myc Targets v2
Inflammatory Response
Complement
UV Response Up
MTORC1 Signaling
UV Response Down
Cholesterol Homeostasis
Glycolysis
Hypoxia
-4
-2
0
2
NES
C
Density
1
0.5
0
1.59
0.81
0
2
4
abs(NES)
variable
siTAZ
siYAP

Supplementary Figure 4: TAZ and YAP1 induce drug tolerance. A Scatter plot showing SOX10 expression compared to YAP1, TAZ, and TEAD1 Chronos CRISPR gene dependency scores for melanoma cell lines (n=62) data obtained from DepMap. Red dots indicate cell lines predicted to have dependence (Chronos score < -1) on WWTR1/TAZ for survival. Cell lines were separated into high and low groups based on a bimodal cutoff of SOX10 expression (> 4.358). Chronos scores were compared between SOX10 High (n=53) and SOX10 low (n=8) groups for YAP1 (p-value = 0.07276), TAZ (p-value = 0.0488), and TEAD1 (p-value = 0.00694). Welch Two Sample t-test. **B** MeWo LacZ, MeWo-YAP1 S127A, and MeWo-TAZ S89A cells were treated -/+ 100 ng/mL doxycycline for 48 hrs. Cell lysates were analyzed by Western blotting with the antibodies indicated. The experiment was repeated independently three times with similar results. **C** Immunofluorescence images of A375, 2.18 SOX10 KO, and 4.21 SOX10 cells stained for TAZ (green), Phalloidin (red), and DAPI (blue). The experiment was performed independently twice, and representative images are shown. Scale bar, 25 µm. **D** MeWo LacZ, MeWo YAP1-S127A, and MeWo TAZ-S89A cells were induced with 100 ng/mL of doxycycline and treated with 50 nM trametinib or vehicle control. Cells were imaged using IncuCyte Live Cell Analysis System. Treatment was renewed every 48-72 hrs. Shown is the mean ± SEM from three independent experiments.

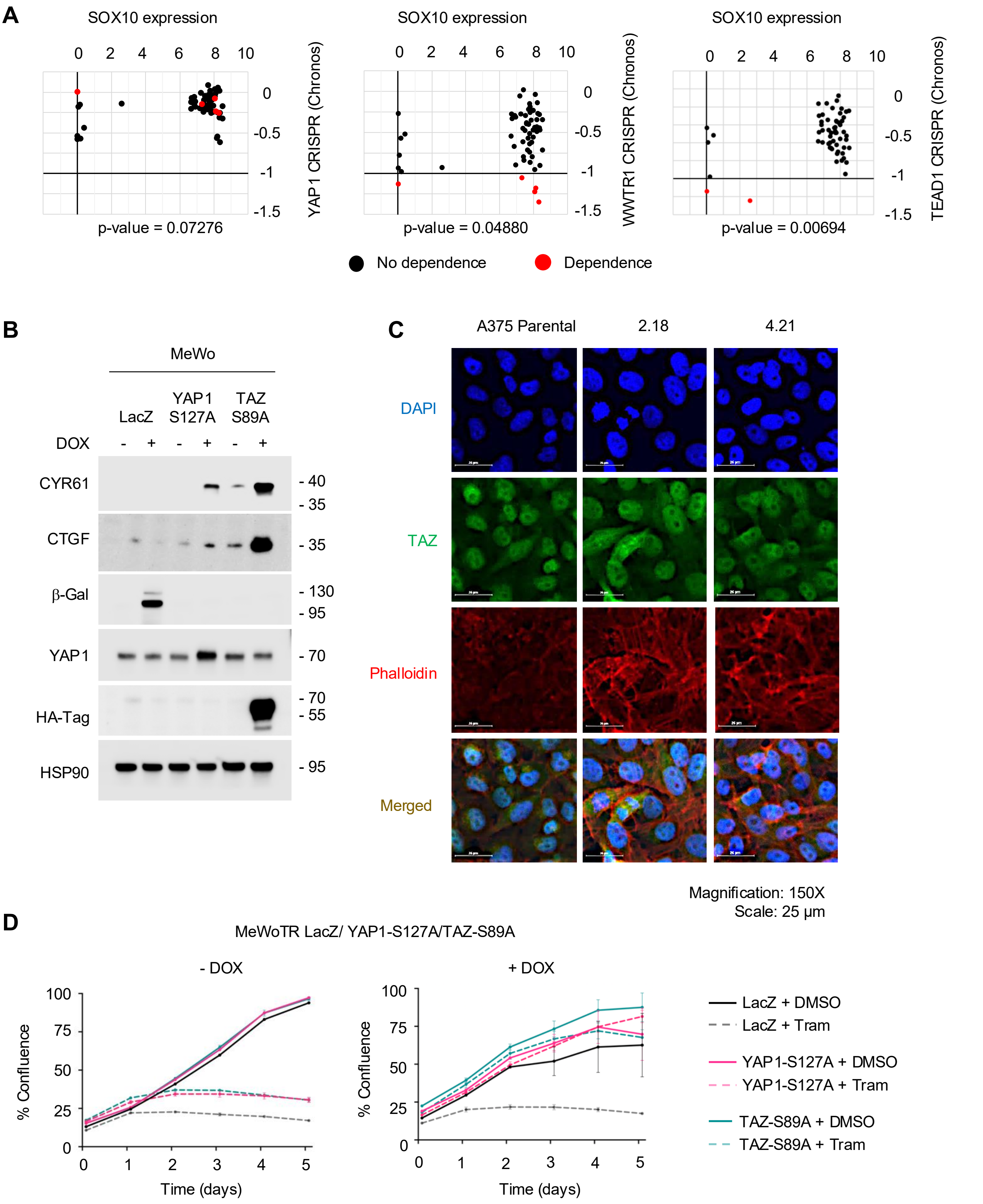
A
SOX10 expression
0 2 4 6 8 10
0
-0.5
-1
-1.5
YAP1 CRISPR (Chronos)
p-value = 0.07276
SOX10 expression
WWTR1 CRISPR (Chronos)
p-value = 0.04880
SOX10 expression
TEAD1 CRISPR (Chronos)
p-value = 0.00694
No dependence
Dependence
B
MeWo
LacZ
YAP1 S127A
TAZ S89A
DOX - + - + - +
CYR61
CTGF
β-Gal
YAP1
HA-Tag
HSP90
- 40
- 35
- 35
- 130
- 95
- 70
- 70
- 55
- 95
C
A375 Parental
2.18
4.21
DAPI
TAZ
Phalloidin
Merged
Magnification: 150X
Scale: 25 μm
D
MeWoTR LacZ/ YAP1-S127A/TAZ-S89A
- DOX
+ DOX
% Confluence
100
75
50
25
0
0 1 2 3 4 5
Time (days)
LacZ + DMSO
LacZ + Tram
YAP1-S127A + DMSO
YAP1-S127A + Tram
TAZ-S89A + DMSO
TAZ-S89A + Tram

Supplementary Figure 5: Characterization of OPN-9652 or OPN-9643 as novel TEAD inhibitors. A NCI-H226 cells are plated at low density in growth media (RPMI-1640, 10% FBS, 1% P/S) in the morning, followed by compound addition in the afternoon when cells are attached to the white flat-bottom 96-well plates (Corning 3610). Cells are incubated in compound-containing media for 5 days before viability (CellTiter Glo, Promega) readout. All experiments are done in duplicates. **B** MSTO-211H cells transfected to express a TEAD luciferase reporter are plated in growth media (RPMI-1640, 10% FBS, 1% P/S) in a black flat-bottom 96-well plate (Corning 3603). On the following day, an equal volume of media is added with titrations of compounds in DMSO and cells are incubated in compound-containing media for 24 hours before viability (CellTiter-Fluor, Promega) and reporter (ONE-Glo, Promega) readout. Reporter luminescence signal is normalized to viability for individual wells for IC_{50} calculations. All experiments are done in duplicates. **C** Depalmitoylated TEAD1 or TEAD4 protein (5µg/well) was incubated with 180 µM of compounds or DMSO vehicle control in 25 mM HEPES pH7.5, 150 mM NaCl, 0.01% Triton X-100 buffer for 1 hr. GloMelt Biotium dye was added before melt curve measurements on a QuantStudio 7 Flex. Delta Tm values were analyzed with a Protein Thermal Shift software (Thermo Fisher). **D** A375 2.18 and A375 4.21 cells were untreated or treated with reagent alone, non-targeting control siRNA, siTEAD1, siTEAD2, siTEAD3, or siTEAD4. After 72 hours, cells were lysed and lysates were analyzed by Western blotting with the antibodies indicated. **E** A375 2.18 and 4.21 SOX10 KO cells were treated with indicated doses of OPN-9652 or OPN-9643 for 48 hours. Cells were lysed and lysates analyzed by Western blotting with the antibodies indicated. **F** A375 TAZ-S89A cells were induced with 100 ng/µL doxycycline, then treated with either 1 µM PLX4270, 35 nM PD0325901 or vehicle control for 24 hrs. Cells were lysed, and lysates were analyzed by Western blotting with the antibodies indicated.

A

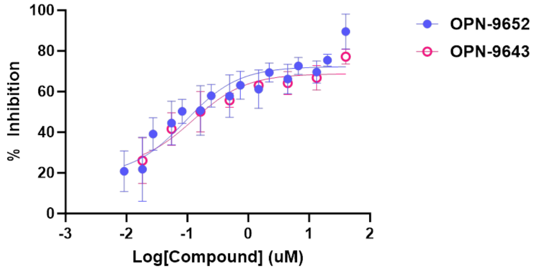

B

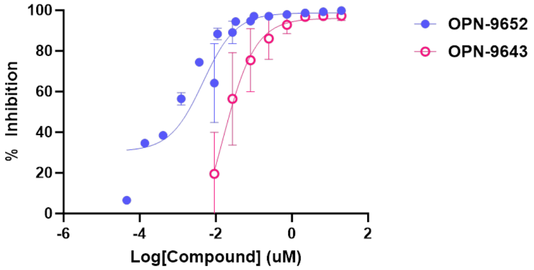

C

OPN-9643 µM	Delta Tm-palmitoylated TEAD4 (°C)	Delta Tm-depalmitoylated TEAD4 (°C)	OPN-9652 µM	Delta Tm-palmitoylated TEAD4 (°C)	Delta Tm-depalmitoylated TEAD4 (°C)
222	-1.77	7.99	222	-1.39	8.05
111	-1.58	7.86	111	-1.39	8.11
55.5	-0.82	8.43	55.5	-1.14	8.62
27.75	-0.50	8.43	27.75	-1.01	8.43
13.75	-0.50	8.37	13.75	-0.69	8.94
6.87	-0.57	0.38	6.87	0.19	0.69
3.43	-0.38	0.88	3.43	-0.76	0.31
1.71	0.19	0.12	1.71	0.12	0.19

D

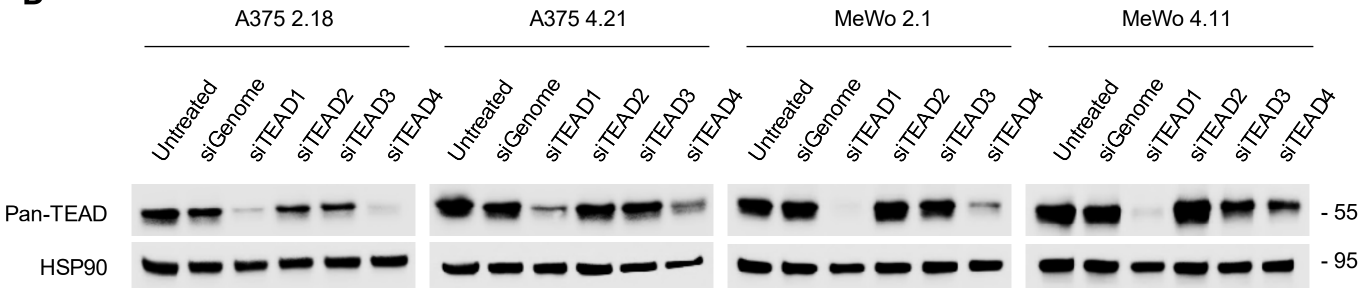

E

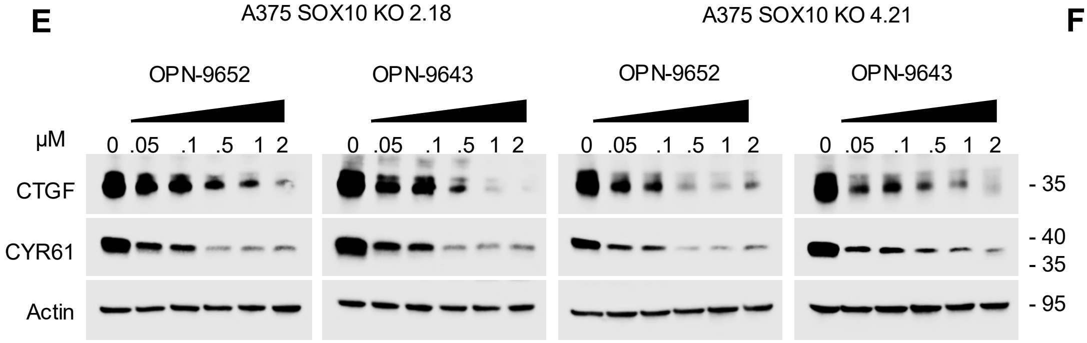

F

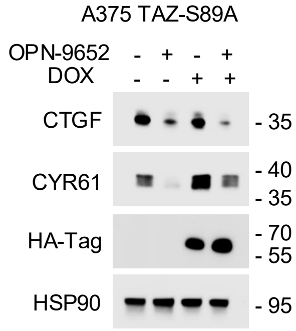

Supplementary Figure 6: RNA-seq analysis of OPN-9652 and OPN-9643 in A375 cells. A A375 crSOX10 #4.21 cells were treated with 1 μM of either OPN-9652, OPN-9643, VT106, or VT107 for 24 hrs and then cells were lysed. Firefly luciferase activity was measured via Dual-Luciferase® Reporter Assay System. The experiment was repeated independently four times with similar results. One-way ANOVA. **B** Heatmap showing GSEA NES for the hallmark gene sets in SOX10 KO cells following treatment of either OPN-9652 or OPN-9643 compared to vehicle-treated cells. NES values are displayed for enriched gene sets using a BHFDR cutoff of 0.05. Shown is the mean from three independent replicates generated for each cell line. **C** Barplot of RNA-seq data showing DKK1, MYC, and TGFβ2 normalized gene expression values in TEADi treated samples compared to DMSO for A375 parental and SOX10 KO cell lines. *$p<0.05$, ***$p<0.001$, ****$p<0.0001$ One-way ANOVA. **D** Barblot of average Normalized Enrichment Scores (NES) across siTAZ, siYAP1, and averaged OPN-9652 and OPN-9643 samples. Displayed signatures were selected from the top and bottom five most significantly changed Hallmark signatures within the TEADi sample group.

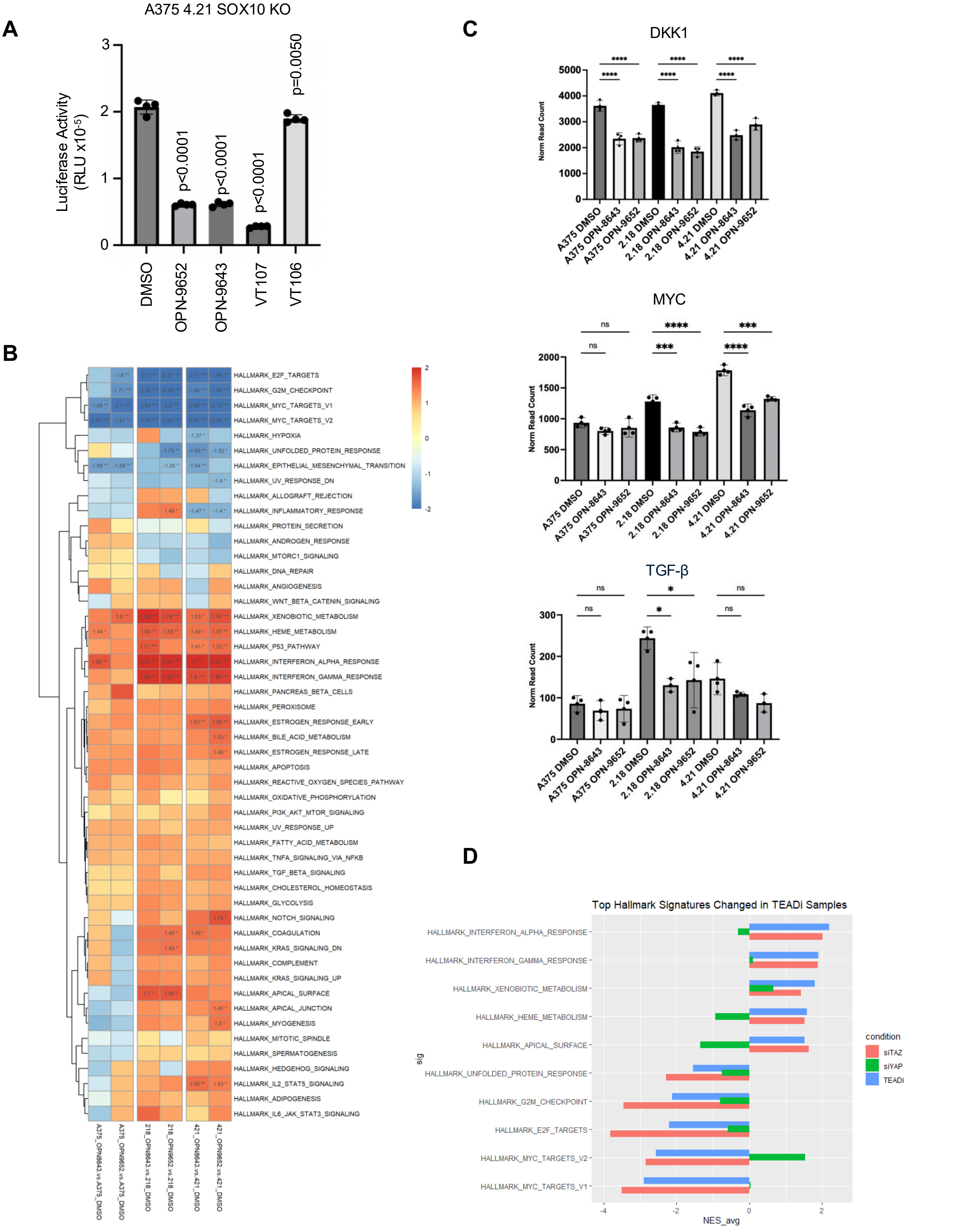
A
A375 4.21 SOX10 KO
Luciferase Activity (RLU x10^-5)
3
2
1
0
p<0.0001
p<0.0001
p<0.0001
p=0.0050
DMSO
OPN-9652
OPN-9643
VT107
VT106
B
HALLMARK_E2F_TARGETS
HALLMARK_G2M_CHECKPOINT
HALLMARK_MYC_TARGETS_V1
HALLMARK_MYC_TARGETS_V2
HALLMARK_HYPOXIA
HALLMARK_UNFOLDED_PROTEIN_RESPONSE
HALLMARK_EPITHELIAL_MESENCHYMAL_TRANSITION
HALLMARK_UV_RESPONSE_DN
HALLMARK_ALLOGRAFT_REJECTION
HALLMARK_INFLAMMATORY_RESPONSE
HALLMARK_PROTEIN_SECRETION
HALLMARK_ANDROGEN_RESPONSE
HALLMARK_MTORC1_SIGNALING
HALLMARK_DNA_REPAIR
HALLMARK_ANGIOGENESIS
HALLMARK_WNT_BETA_CATENIN_SIGNALING
HALLMARK_XENOBIOTIC_METABOLISM
HALLMARK_HEME_METABOLISM
HALLMARK_P53_PATHWAY
HALLMARK_INTERFERON_ALPHA_RESPONSE
HALLMARK_INTERFERON_GAMMA_RESPONSE
HALLMARK_PANCREAS_BETA_CELLS
HALLMARK_PEROXISOME
HALLMARK_ESTROGEN_RESPONSE_EARLY
HALLMARK_BILE_ACID_METABOLISM
HALLMARK_ESTROGEN_RESPONSE_LATE
HALLMARK_APOPTOSIS
HALLMARK_REACTIVE_OXYGEN_SPECIES_PATHWAY
HALLMARK_OXIDATIVE_PHOSPHORYLATION
HALLMARK_PI3K_AKT_MTOR_SIGNALING
HALLMARK_UV_RESPONSE_UP
HALLMARK_FATTY_ACID_METABOLISM
HALLMARK_TNFA_SIGNALING_VIA_NFKB
HALLMARK_TGF_BETA_SIGNALING
HALLMARK_CHOLESTEROL_HOMEOSTASIS
HALLMARK_GLYCOLYSIS
HALLMARK_NOTCH_SIGNALING
HALLMARK_COAGULATION
HALLMARK_KRAS_SIGNALING_DN
HALLMARK_COMPLEMENT
HALLMARK_KRAS_SIGNALING_UP
HALLMARK_APICAL_SURFACE
HALLMARK_APICAL_JUNCTION
HALLMARK_MYOGENESIS
HALLMARK_MITOTIC_SPINDLE
HALLMARK_SPERMATOGENESIS
HALLMARK_HEDGEHOG_SIGNALING
HALLMARK_IL2_STAT5_SIGNALING
HALLMARK_ADIPOGENESIS
HALLMARK_IL6_JAK_STAT3_SIGNALING
A375_OPN8643.vs.A375_DMSO
A375_OPN9652.vs.A375_DMSO
218_OPN8643.vs.218_DMSO
218_OPN9652.vs.218_DMSO
421_OPN8643.vs.421_DMSO
421_OPN9652.vs.421_DMSO
C
DKK1
Norm Read Count
MYC
TGF-β
ns
A375 DMSO
A375 OPN-8643
A375 OPN-9652
2.18 DMSO
2.18 OPN-8643
2.18 OPN-9652
4.21 DMSO
4.21 OPN-8643
4.21 OPN-9652
D
Top Hallmark Signatures Changed in TEADi Samples
HALLMARK_INTERFERON_ALPHA_RESPONSE
HALLMARK_INTERFERON_GAMMA_RESPONSE
HALLMARK_XENOBIOTIC_METABOLISM
HALLMARK_HEME_METABOLISM
HALLMARK_APICAL_SURFACE
HALLMARK_UNFOLDED_PROTEIN_RESPONSE
HALLMARK_G2M_CHECKPOINT
HALLMARK_E2F_TARGETS
HALLMARK_MYC_TARGETS_V2
HALLMARK_MYC_TARGETS_V1
sig
NES_avg
condition
siTAZ
siYAP
TEADi

Supplementary Figure 7: OPN-9652 enhances BRAFi + MEKi effects in SOX10 KO melanoma. A A375 crSOX10 #2.18, and A375 crSOX10 #4.21 cells were treated with 2 µM of either OPN-9652 or OPN-9643 and imaged using IncuCyte Live Cell Analysis System. Treatment was renewed every 48-72 hrs. Cell growth was determined as percent plate coverage. Shown is the mean ± SEM from three independent experiments. Boxplot graphs of the log-transformed plate-specific AUC for statistical analysis of data in 3C Log-transformed plate-specific AUCs were analyzed in a two-way ANOVA model with the nominal effect of dose, cell line (A375 SOX10 KO #2.18 and #4.21), and their interaction by two-way ANOVA. **B** Boxplot graphs of the log-transformed plate-specific AUC for statistical analysis of data in 3A Log-transformed plate-specific AUCs were analyzed in a two-way ANOVA model with the nominal effect of dose, cell line (A375 SOX10 KO #2.18 and #4.21), and their interaction. Mean AUC ratio, 95% confidence interval, and p-values are indicated in table. **C** Representative phase contrast images and Incucyte mask of MeWo crSOX10 #2.1 cells treated with 50 nM of Trametinib, and 2 µM of either OPN-9652 or OPN-9643. Treatment was renewed every 48-72 hrs, and images were taken from the same well over 4 different time points.

A

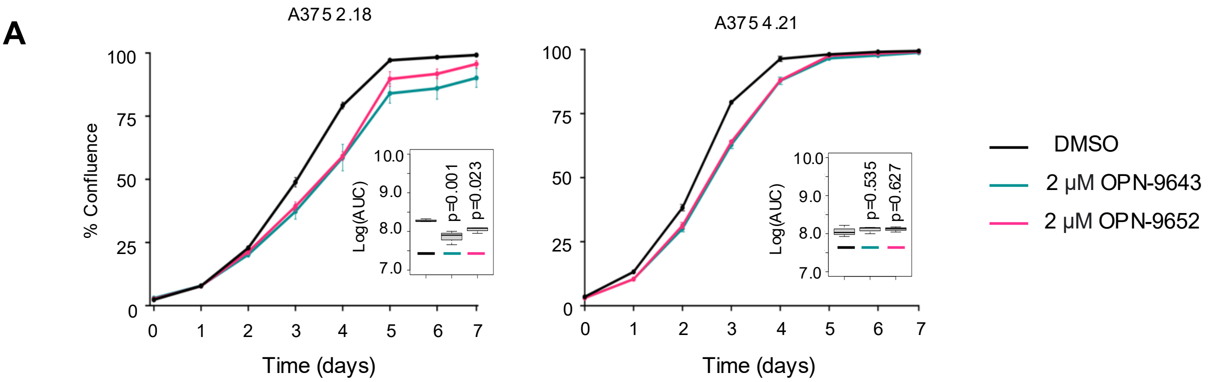

B

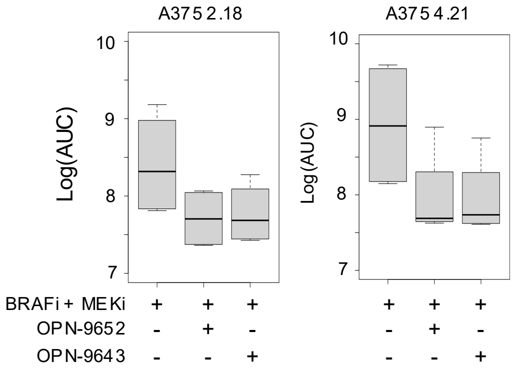

Comparison of BRAFi + MEKi vs:	Mean AUC Ratio	95% conf. interval		P-value
		Lower	Upper	
BRAFi + MEKi + OPN-9643	0.44	0.24	0.81	0.011
BRAFi + MEKi + OPN-9652	0.45	0.24	0.83	0.013

C

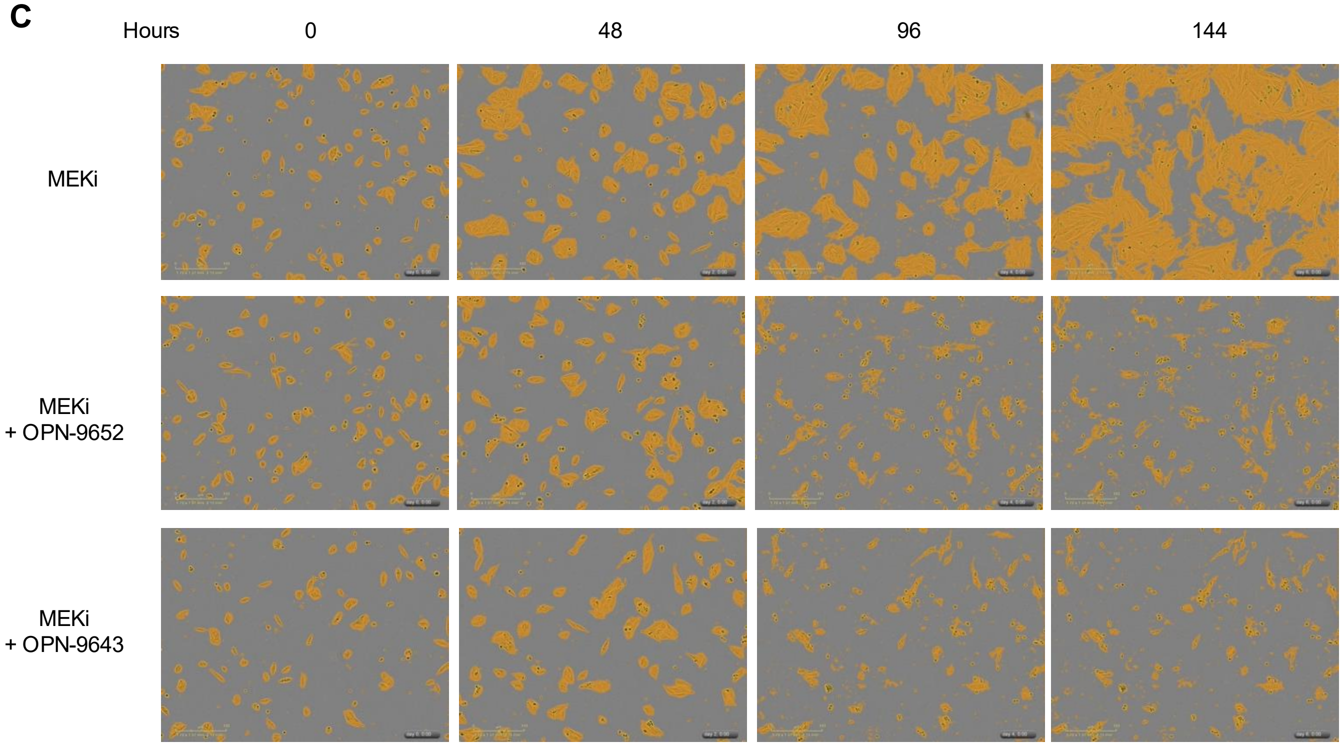

Supplementary Figure 8: OPN-9652 in combination with BRAFi + MEKi in melanoma 3D spheroid models. A Quantification of cell proliferation using an EdU incorporation assay. Bar plot depicting the percentage of EdU-positive cells in MeWo 4.11 following treatment of 50 nm Trametinib, 2µM OPN-9652, or combination for 48 hrs. Cells were incubated with 10 µM EdU for 16 hrs, followed by fixation and Click-iT EdU staining to detect proliferating cells. Data represent the mean ± SEM from technical triplicates. Statistical significance was determined using One-way Anova,****p < 0.001**. B** Representative images of 3D tumor spheroids for A375 SOX10 KO melanoma cell lines, #2.18 and #4.21, following 96 hrs of treatment using either BRAFi + MEKi (1 µM PLX4270 + 35 nM PD0325901), TEADi (2 µM OPN-9643) or triple combination therapy. 3D spheroids were stained with calcein-AM (7 µM) for cell viability evaluation. 3D spheroids were stained with propidium iodide (10 µg/mL) for cell death evaluation. Scale bars: 1000 µm. Magnification: 40X. **C** Quantification bar graph of Outgrowth Area from Fig. 7B. Data are shown as mean ± SEM (n=3). p values by unpaired t-test. **D** Quantification bar graph of cell death from Fig. 7B. Data are shown as mean ± SEM (n=3). p values were derived by unpaired t-test.

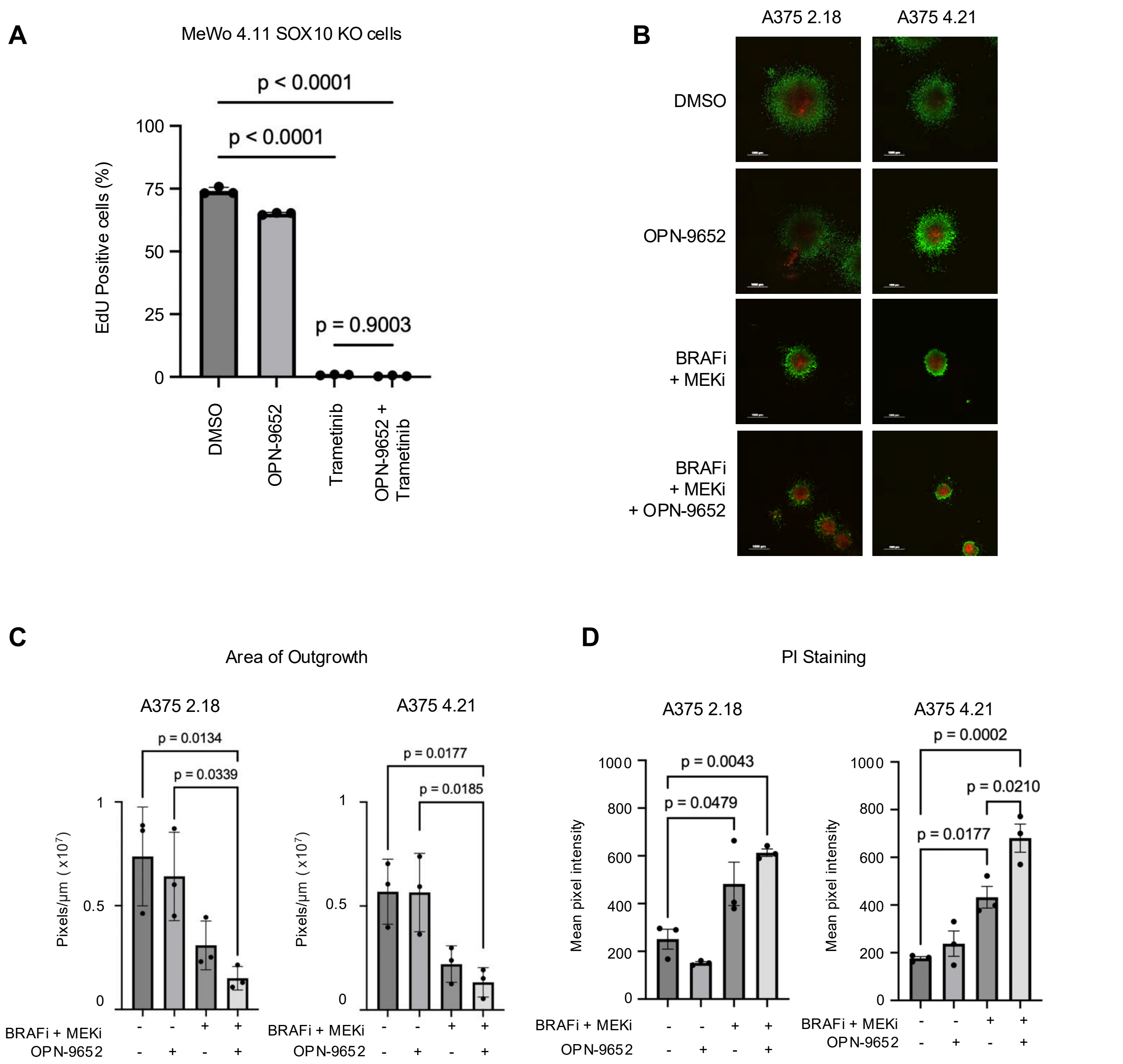
A
MeWo 4.11 SOX10 KO cells
EdU Positive cells (%)
p < 0.0001
p < 0.0001
p = 0.9003
DMSO
OPN-9652
Trametinib
OPN-9652 + Trametinib
B
A375 2.18
A375 4.21
DMSO
OPN-9652
BRAFi + MEKi
BRAFi + MEKi + OPN-9652
C
Area of Outgrowth
A375 2.18
A375 4.21
Pixels/μm (x10^7)
p = 0.0134
p = 0.0339
p = 0.0177
p = 0.0185
BRAFi + MEKi
OPN-9652
D
PI Staining
A375 2.18
A375 4.21
Mean pixel intensity
p = 0.0043
p = 0.0479
p = 0.0002
p = 0.0210
p = 0.0177
BRAFi + MEKi
OPN-9652

Supplementary Figure 9: OPN-9652 in combination with BRAFi + MEKi extends survival in YUMM1.7 compared to BRAFi + MEKi alone. A Individual tumor growth graph comparing treatment arms of A375 xenograft treated with PLX4720 (200 PPM), PD0325901 (7 PPM) alone (6 mice) or in combination with OPN-9652 (50 mg/kg) (7 mice). **B** 1014, YUMM1.1, and YUMM1.7 cell lysates were analyzed by Western blotting with the antibodies indicated. The experiment was repeated independently three times with similar results. **C** YUMM1.7 cells were injected into NSG mice. Mouse survival curves comparing treatment arms of YUMM1.7 xenografts treated with PLX4720 (200 PPM), PD0325901 (7 PPM) alone (4 mice) or in combination with OPN-9652 (50 mg/kg) (5 mice) indicating time for tumor to reach 1000 mm^3. Censored mice were due to unexplained deaths. **D** Individual tumor growth graphs comparing treatment arms of YUMM1.7 xenograft treated with PLX4720 (200 PPM), PD0325901 (7 PPM) alone (4 mice) or in combination with OPN-9652 (50 mg/kg; 5 mice).

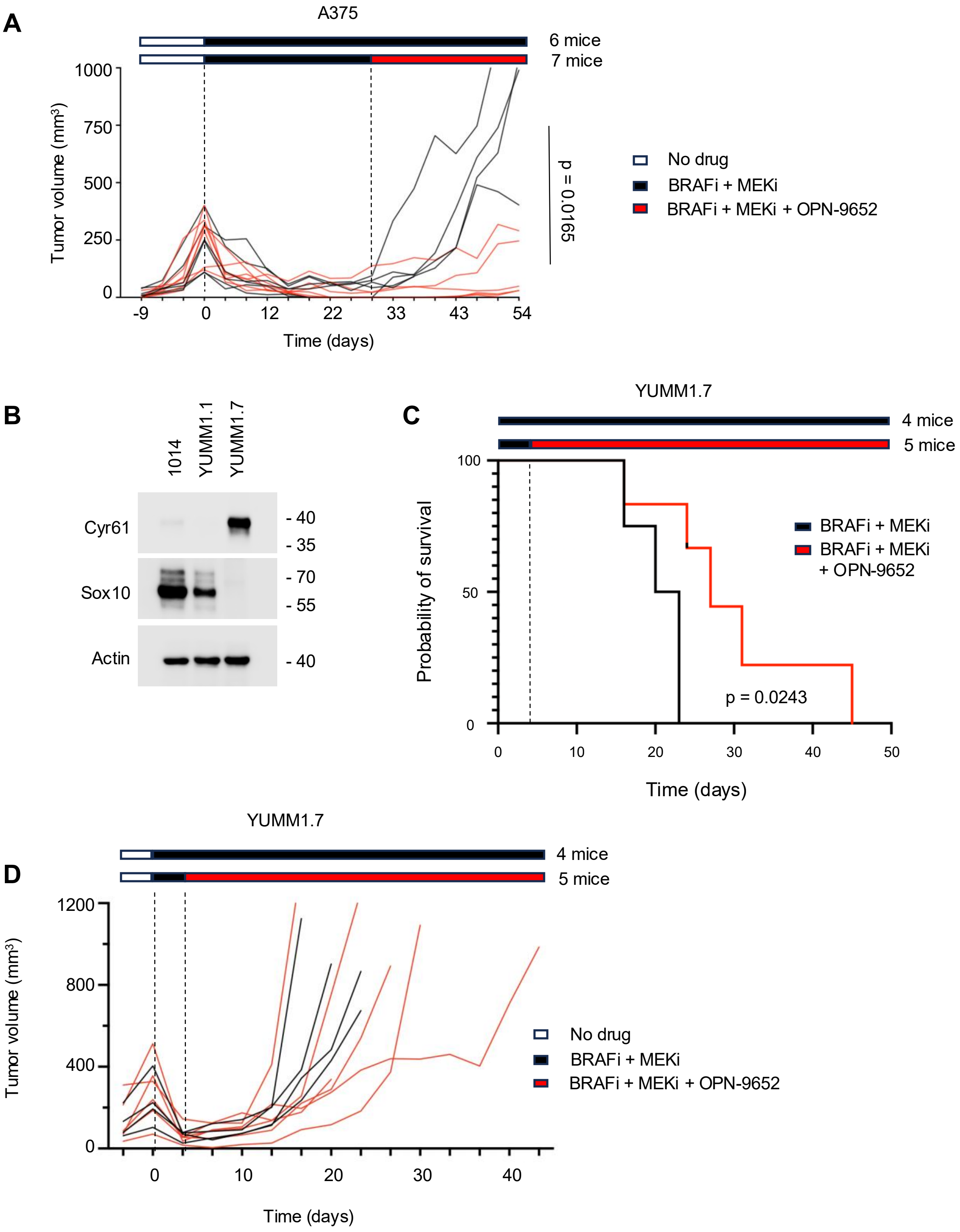
A
A375
6 mice
7 mice
1000
750
500
250
0
Tumor volume (mm³)
-9
0
12
22
33
43
54
Time (days)
p = 0.0165
No drug
BRAFi + MEKi
BRAFi + MEKi + OPN-9652
B
1014
YUMM1.1
YUMM1.7
Cyr61
Sox10
Actin
- 40
- 35
- 70
- 55
- 40
C
YUMM1.7
4 mice
5 mice
100
50
0
Probability of survival
0
10
20
30
40
50
Time (days)
BRAFi + MEKi
BRAFi + MEKi
+ OPN-9652
p = 0.0243
D
YUMM1.7
4 mice
5 mice
1200
800
400
0
Tumor volume (mm³)
0
10
20
30
40
Time (days)
No drug
BRAFi + MEKi
BRAFi + MEKi + OPN-9652

Supplemental Note

Supplemental Results to:

Targeting TAZ-TEAD in minimal residual disease enhances the duration of targeted therapy in melanoma models

Synthesis and characterization of TEAD inhibitors (continued).

Synthesis of tert-butyl 7-(4,4,5,5-tetramethyl-1,3,2-dioxaborolan-2-yl)-3,4-dihydroisoquinoline-2(1H)-carboxylate (3):

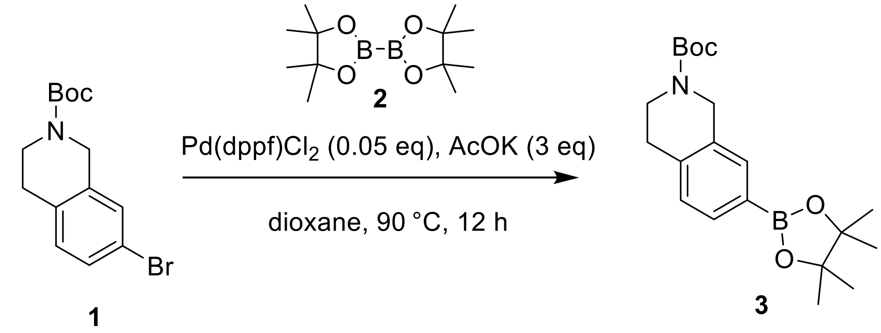

tert-butyl 7-bromo-3,4-dihydro-1*H*-isoquinoline-2-carboxylate (**1**) (100 g, 320.31 mmol, 1 eq), anhydrous dioxane (1 L), 4,4,5,5-tetramethyl-2-(4,4,5,5-tetramethyl-1,3,2-dioxaborolan-2-yl)-1,3,2-dioxaborolane (**2**) (122 g, 480.46 mmol, 1.5 eq), and AcOK (94.31 g, 960.92 mmol, 3 eq) were added. The resultant mixture was purged with N_2 for 5 minutes and then Pd(dppf)Cl_2 (11.72 g, 16.02 mmol, 0.05 eq) was added. The mixture was purged with N_2 for another 5 minutes and then stirred at 90°C for 12 hours before cooling to room temperature. Liquid chromatography-mass spectrometry (LCMS) indicated the reaction was completed. The reaction was performed in three batches in parallel and the mixtures of three batches were poured into water (3 L). The mixture was extracted with ethyl acetate (2 L x 2). The combined organic extracts were dried over anhydrous Na_2SO_4, filtered, and concentrated by evaporation under reduced pressure to give the crude product, which was purified by column chromatography (SiO_2, eluent: petroleum ether: ethyl acetate = 100: 1 to 10: 1) to afford the title compound **3** (344 g, 99 % yield).

LC-MS Electrospray ionization (ESI): RT = 1.73 min, mass calculated. for $C_{20}H_{30}BNO_4$ 359.23 m/z, found 304.04 $[M-56+H]^+$.

Synthesis of tert-butyl 7-hydroxy-3,4-dihydroisoquinoline-2(1H)-carboxylate (4):

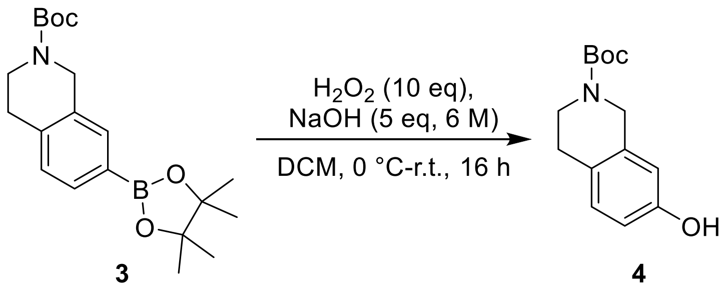

3 **4**

NaOH (6 M, 200 mL, 5 eq) was added drop-wise to a solution of *tert*-butyl 7-(4,4,5,5-tetramethyl-1,3,2-dioxaborolan-2-yl)-3,4-dihydroisoquinoline-2(1*H*)-carboxylate (86 g, 239.38 mmol, 1 eq) in anhydrous dichloromethane (1 L) at 0°C. The resultant mixture was stirred at 0°C for 10 minutes before treating with H_2O_2 (280 g, 2.47 mol, 237.29 mL, 30% purity, 10.32 eq) in portions at 0°C under N_2. The resultant mixture was stirred for 16 hours with gradual warming to room temperature. LCMS indicated the reaction completed. The mixture was cooled to 0°C. The reaction was performed in four batches in parallel and the reaction mixtures of four batches were poured into a saturated aqueous $Na_2S_2O_3$ (3 L) at 0°C. The resultant mixture was then stirred for another 1 hour. The reaction was checked by potassium iodide-starch test paper to see if the residual H_2O_2 was destroyed. Next, the mixture was adjusted to pH 7 with 6 M HCl. Two phases were separated. The aqueous layer was extracted with dichloromethane (1.5 L x 2). The combined organic layer was dried over anhydrous Na_2SO_4, filtered, and concentrated by evaporation under reduced pressure to give the crude product, which was purified by column chromatography (SiO_2, eluent: petroleum ether: ethyl acetate = 1: 0 to 6: 1) to afford the title compound (195 g, 84 %).

LC-MS (ESI): RT = 1.09 min, mass calculated. for $C_{14}H_{19}NO_3$ 249.14 m/z, found 194.00 $[M-56+H]^+$. Method: the gradient was 30-100% B in 1.5 min, 100-100% B in 1 min, 100-30% B in 0.01 min (0.3 mL/min flow rate). Mobile phase A was 0.01% CF_3COOH in water, mobile phase B was 0.01% CF_3COOH in CH_3CN. The column used for the chromatography was a Chromolith Flash waters-BEH-C18 1.7 μm, 2.1 x 50 mm column. Detection methods were diode array (waters-UPLC-PDA) as well as positive electrospray ionization (waters-SQD-MS).

^1H NMR (400 MHz, DMSO-d_6) δ 9.21 (s, 1H), 6.93 (d, *J* = 8.2 Hz, 1H), 6.57 (dd, *J* = 2.6, 8.2 Hz, 1H), 6.51 (s, 1H), 4.38 (s, 2H), 3.49 (t, *J* = 5.9 Hz, 2H), 2.63 (t, *J* = 5.8 Hz, 2H), 1.42 (s, 9H)

Synthesis of tert-butyl 7-(4-(trifluoromethyl)phenoxy)-3,4-dihydroisoquinoline-2(1H)-carboxylate (6):

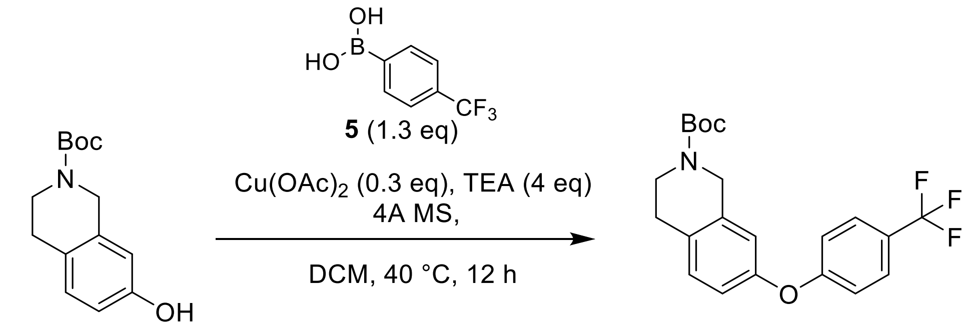

4 **6**

Tert-butyl 7-hydroxy-3,4-dihydroisoquinoline-2(1*H*)-carboxylate (100 g, 401.12 mmol, 1 eq), (4-(trifluoromethyl)phenyl)boronic acid (100 g, 526.52 mmol, 1.31 eq), anhydrous dichloromehane (1 L), TEA (162.35 g, 1.60 mol, 4 eq), and 4Å molecular sieve (20 g) were added to three-necked round-bottomed flask equipped with stirrer, condensing tube, and thermometer. The resultant mixture was purged with O_2 for 5 minutes and then $Cu(OAc)_2$ (21.86 g, 120.33 mmol, 0.3 eq) was added. The mixture was purged with O_2 for another 5 minutes and then stirred at 40 °C for 12 hours under O_2 (15 psi) before cooling to room-temperature. LCMS indicated the reaction completed. The suspension was filtered through a pad of Celite® and the pad was washed with dichloromethane (500 mL). The filtrate was diluted with water (1 L) and the two phases were separated. The aqueous layer was extracted with dichloromethane (1 L x 2). The combined organic layer was dried over anhydrous Na_2SO_4, filtered, and concentrated by evaporation under reduced pressure to give the crude product, which was purified by column chromatography (SiO_2, eluent: petroleum ether: ethyl acetate = 1:0 to 10:1) to afford the title compound (117 g, 74%).

LC-MS (ESI): RT = 2.11 min, mass calculated. for $C_{21}H_{22}F_3NO_3$ 393.16 m/z, found 337.96 $[M-56+H]^+$. Method: the gradient was 30-100% B in 1.5 min, 100-100% B in 1 min, 100-30% B in 0.01 min (0.3 mL/min flow rate). Mobile phase A was 0.01% CF_3COOH in water, mobile phase B was 0.01% CF_3COOH in CH_3CN. A Chromolith Flash waters-BEH-C18 1.7 μm, 2.1 x 50 mm column was used for the chromatography. Detection methods are diode array (waters-UPLC-PDA) as well as positive electrospray ionization (waters-SQD-MS).

^{1}H NMR (400MHz, DMSO-d_6) δ 7.74 - 7.69 (m, 2H), 7.24 (d, *J* = 8.3 Hz, 1H), 7.14 - 7.06 (m, 2H), 7.00 (s, 1H), 6.97 - 6.92 (m, 1H), 4.49 (s, 2H), 3.56 (t, *J* = 5.9 Hz, 2H), 2.78 (t, *J* = 5.7 Hz, 2H), 1.42 (s, 9H).

Synthesis of 7-(4-(trifluoromethyl)phenoxy)-1,2,3,4-tetrahydroisoquinoline hydrochloride (7):

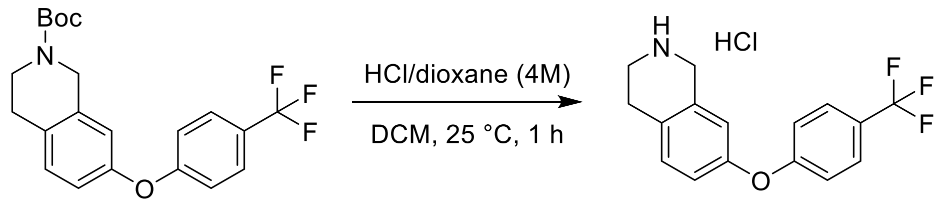

6 7

HCl/dioxane (4M, 250 mL) was added to a solution consisting of *tert*-butyl 7-(4-(trifluoromethyl)phenoxy)-3,4-dihydroisoquinoline-2(1*H*)-carboxylate (68 g, 172.85 mmol, 1 eq) in anhydrous dichloromehane (700 mL). The resultant solution was stirred at 25°C for 1 hr. LCMS indicated the reaction completed. The suspension was concentrated by evaporation under reduced pressure to give the crude product (53 g, crude).

[Note: 14.91 g of the crude product was firstly triturated with petroleum ether: ethyl acetate = 5: 1 (100 mL) and the suspension was isolated via filtration. The filter cake washed with petroleum ether (50 mL) before drying under reduced pressure. The obtained solid was further triturated with petroleum ether: ethyl acetate = 3: 1 (100 mL) (for two times). The suspension was isolated via filtration. The filter cake was washed with petroleum ether (50 mL) before drying under reduced pressure to afford the pure title compound (7.66 g).

LC-MS (ESI): RT = 3.33 min, mass calculated for $C_{16}H_{14}F_3NO$ 293.10 m/z, found 294.0 $[M+H]^+$. Method: the gradient was 5-95% B in 5 min, 95-95% B in 1.0 min, 95-5% B in 0.01 min (1.0 mL/min flow rate). Mobile phase A was 0.01% CF_3COOH in water, mobile phase B was 0.01% CF_3COOH in CH_3CN. A Chromolith Flash waters-BEH-C18 1.7um, 2.1 x 50 mm column was utilized. Detection methods were diode array (waters-UPLC-PDA) as well as positive electrospray ionization (waters-SQD-MS).

^1H NMR (400MHz, $CDCl_3$) δ 10.20 (br s, 1H), 7.65 - 7.53 (m, 2H), 7.22 - 7.15 (m, 1H), 7.07 - 6.98 (m, 2H), 6.97 - 6.90 (m, 1H), 6.86 - 6.77 (m, 1H), 4.43 - 4.24 (m, 2H), 3.55 - 3.41 (m, 2H), 3.25 - 3.09 (m, 2H)

Synthesis of 1-(7-(4-(trifluoromethyl)phenoxy)-3,4-dihydroisoquinolin-2(1H)-yl)prop-2-en-1-one (OPN9643):

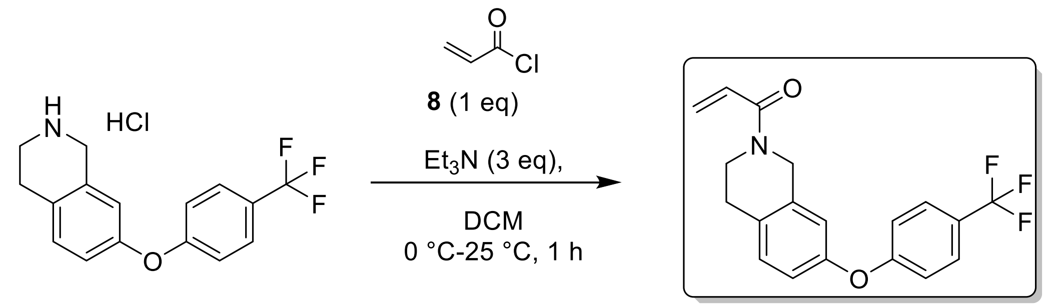

7 **OPN9643**

Acryloyl chloride (9.06 g, 100.08 mmol, 8.16 mL, 1 eq) was drop-wise to a solution consisting of 7-(4-(trifluoromethyl)phenoxy)-1,2,3,4-tetrahydroisoquinoline (33 g, 100.08 mmol, 1 eq, HCl), TEA (30.38 g, 300.23 mmol, 41.79 mL, 3 eq), and anhydrous dichloromethane (300 mL) at 0 °C. The resultant solution was stirred for 1 hour with gradual warming to 25°C. LCMS indicated the reaction completed. The reaction mixture was quenched with saturated NH_4Cl (100 mL). The mixture was extracted with dichloromethane (500 mL). The combined organic extract was washed with water (200 mL x 2), dried over anhydrous Na_2SO_4, filtered, and concentrated by evaporation under reduced pressure to give the crude product, which was purified by flash column (eluent: petroleum ether: ethyl acetate = 1:0 to 2:1). The reaction was performed in two batches in parallel and total product (24 g, 97% purity) as a light green solid was triturated with petroleum ether: ethyl acetate = 5:1 (250 mL) and the suspension was isolated via filtration. The filter cake was washed with petroleum ether (150 mL) before drying under reduced pressure to afford the title compound (20.78 g, 89%).

LC-MS (ESI): RT = 3.49 min, mass calculated. for $C_{19}H_{16}F_3NO_2$ 347.11 m/z, found 348.0 $[M+H]^+$. Method: the gradient was 30-95% B in 5 min, 95-95% B in 1.0 min, 95-30% B in 0.01 min. (1.0 mL/min flow rate). Mobile phase A was 0.01% CF_3COOH in water, mobile phase B was 0.01% CF_3COOH in CH_3CN. A Chromolith Flash waters-BEH-C18 1.7 μm, 2.1 x 50 mm column was used for chromatography. Detection methods are diode array (waters-UPLC-PDA) as well as positive electrospray ionization (waters-SQD-MS).

^{1}H NMR (400MHz, DMSO-d_6) δ 7.79 - 7.68 (m, 2H), 7.26 (d, *J* = 8.6 Hz, 1H), 7.18 - 7.09 (m, 2H), 7.08 - 6.94 (m, 2H), 6.94 - 6.79 (m, 1H), 6.15 (dd, *J* = 2.3, 16.6 Hz, 1H), 5.78 - 5.65 (m, 1H), 4.82 - 4.62 (m, 2H), 3.88 - 3.69 (m, 2H), 2.93 - 2.76 (m, 2H).

Synthesis of *tert*-Butyl 7-(3-fluoro-4-(trifluoromethyl)phenoxy)-3,4-dihydroisoquinoline-2(1*H*)-carboxylate (10):

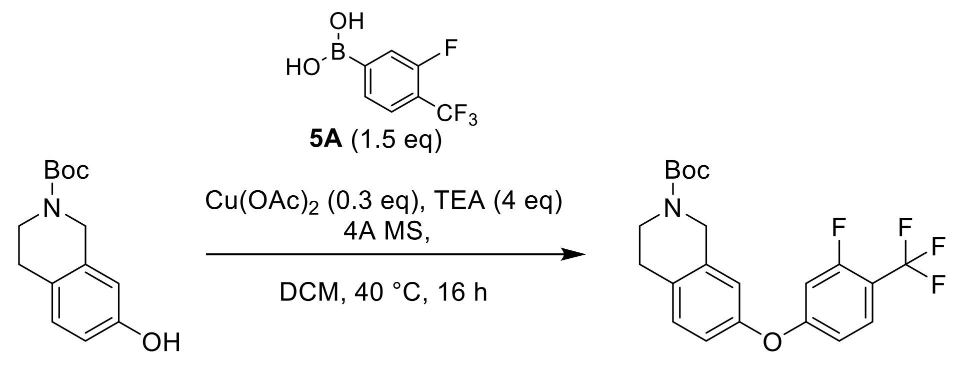

4 **10**

Tert-butyl 7-hydroxy-3,4-dihydroisoquinoline-2(1*H*)-carboxylate (37 g, 148.41 mmol, 1 eq), (3-fluoro-4-(trifluoromethyl)phenyl)boronic acid (46.29 g, 222.62 mmol, 1.5 eq), anhydrous dichloromethane (500 mL), TEA (60.07 g, 593.65 mmol, 82.63 mL, 4 eq), and 4Å molecular sieve (8 g) were added to a 1 L three-necked round-bottomed flask equipped with stirrer, condensing tube, and thermometer. The resultant mixture was purged with O_2 for 5 minutes and then $Cu(OAc)_2$ (8.09 g, 44.52 mmol, 0.3 eq) was added. The mixture was purged with O_2 for another 5 minutes and then stirred at 40 °C for 16 hours under O_2 (15 psi) before cooling to room temperature. LCMS indicated the reaction was completed. The reaction was performed in two batches in parallel and the reaction mixtures of two batches were filtered through a pad of Celite® and the pad was washed with dichloromethane (500 mL). The filtrate was diluted with water (800 mL) and the two phases were separated. The aqueous layer was extracted with dichloromethane (800 mL x 2). The combined organic layer was dried over anhydrous Na_2SO_4, filtered, and concentrated by evaporation under reduced pressure to give the crude product, which was purified by column chromatography (SiO_2, eluent: petroleum ether: ethyl acetate = 1: 0 to 11: 1) to afford the title compound (33 g, 27 %).

LC-MS (ESI): RT = 1.54 min, mass calculated for $C_{21}H_{21}F_4NO_3$ 411.15 m/z, found 356.6 $[M-56+H]^+$. Method: the gradient was 30-100% B in 1.5 min, 100-100% B in 1.0 min, 100-30% B in 0.01 min. (0.3 mL/min flow rate). Mobile phase A was 0.01% CF_3COOH in water, mobile phase B was 0.01% CF_3COOH in CH_3CN. The column used for the chromatography is a Chromolith Flash waters-BEH-C18 1.7 μm, 2.1 x 50 mm column. Detection methods are diode array (waters-UPLC-PDA) as well as positive electrospray ionization (waters-SQD-MS).

^1H NMR (400MHz, $CDCl_3$) δ 7.55 - 7.49 (m, 1H), 7.21 - 7.16 (m, 1H), 6.91 - 6.87 (m, 1H), 6.85 - 6.82 (m, 1H), 6.82 - 6.77 (m, 1H), 6.76 - 6.69 (m, 1H), 4.57 (s, 2H), 3.72 - 3.65 (m, 2H), 2.90 - 2.81 (m, 2H), 1.51 - 1.49 (m, 9H).

Synthesis of 7-(3-fluoro-4-(trifluoromethyl)phenoxy)-1,2,3,4-tetrahydroisoquinoline hydrochloride (11):

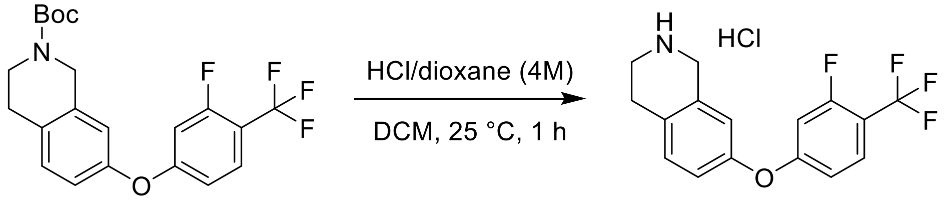

10 **11**

HCl/dioxane (4M, 150 mL) was added to a solution consisting of *tert*-butyl 7-[3-fluoro-4-(trifluoromethyl)phenoxy]-3,4-dihydro-1*H*-isoquinoline-2-carboxylate (33 g, 80.22 mmol, 1 eq) in anhydrous dichloromethane (400 mL). The resultant solution was stirred at 25 °C for 1 hr. LCMS indicated the reaction completed. The suspension was concentrated by evaporation under reduced pressure to give the crude product **11** (27 g, crude) as a yellow solid, which was used directly in the next step without further purification.

Synthesis of 1-(7-(3-fluoro-4-(trifluoromethyl)phenoxy)-3,4-dihydroisoquinolin-2(1H)-yl)prop-2-en-1-one (OPN9652):

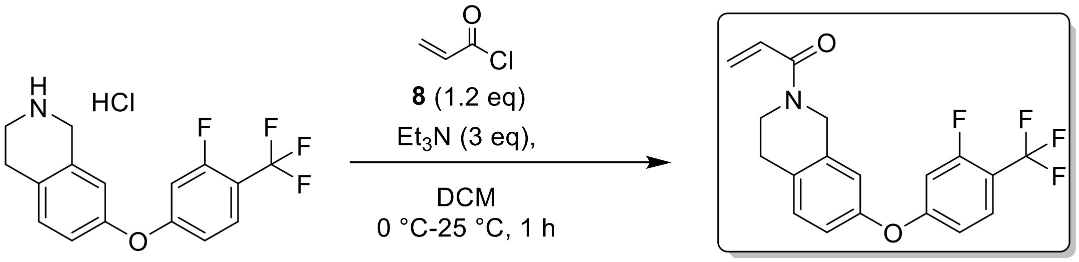

OPN9652

11

Acryloyl chloride (8.43 g, 93.17 mmol, 7.60 mL, 1.2 eq) was drop-wise to a solution consisting of 7-[3-fluoro-4-(trifluoromethyl)phenoxy]-1,2,3,4-tetrahydroisoquinoline (27 g, 77.65 mmol, 1 eq, HCl), TEA (23.58 g, 232.95 mmol, 32.43 mL, 3 eq) and anhydrous dichloromethane (300 mL) at 0 °C. The resultant solution was stirred for 1 hr with gradual warming to 25 °C. LCMS indicated the reaction was completed. The reaction mixture was quenched with saturated NH_4Cl (100 mL). The mixture was extracted with dichloromethane (500 mL). The combined organic extract was washed with water (200 mL x 2), dried over anhydrous Na_2SO_4, filtered, and concentrated by evaporation under reduced pressure to give the crude product, which was purified by flash column (eluent: petroleum ether: ethyl acetate = 1:0 to 2:1) to afford the title compound (18.83 g, 66%).

LC-MS (ESI): RT = 2.57 min, mass calculated for $C_{19}H_{15}F_4NO_2$ 365.10 m/z, found 366.0 $[M+H]^+$. Method: the gradient was 50-95% B in 5 min, 95-95% B in 1.0 min, 95-50% B in 0.01 min. (1.0

mL/min flow rate). Mobile phase A was 0.01% CF_3COOH in water, mobile phase B was 0.01% CF_3COOH in CH_3CN. The column used for the chromatography is a Chromolith Flash waters-BEH-C18 1.7 μm, 2.1 x 50 mm column. Detection methods are diode array (waters-UPLC-PDA) as well as positive electrospray ionization (waters-SQD-MS).

^1H NMR (400MHz, DMSO-d_6) δ 7.81 - 7.69 (m, 1H), 7.28 (d, J = 8.6 Hz, 1H), 7.21 - 6.98 (m, 3H), 6.95 - 6.78 (m, 2H), 6.16 (dd, J = 2.3, 16.6 Hz, 1H), 5.72 (dd, J = 2.3, 10.5 Hz, 1H), 4.81 - 4.65 (m, 2H), 3.86 - 3.72 (m, 2H), 2.93 - 2.76 (m, 2H).

Spectral Traces of OPN9643 and OPN9652

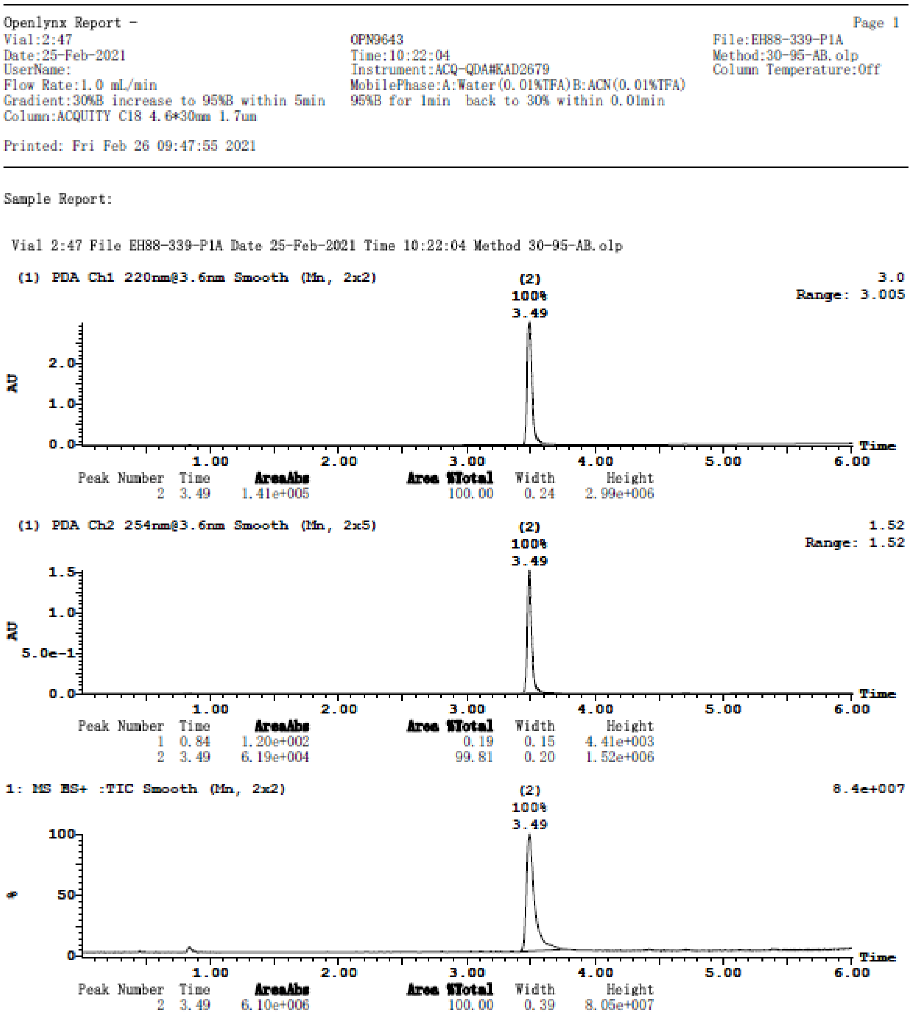

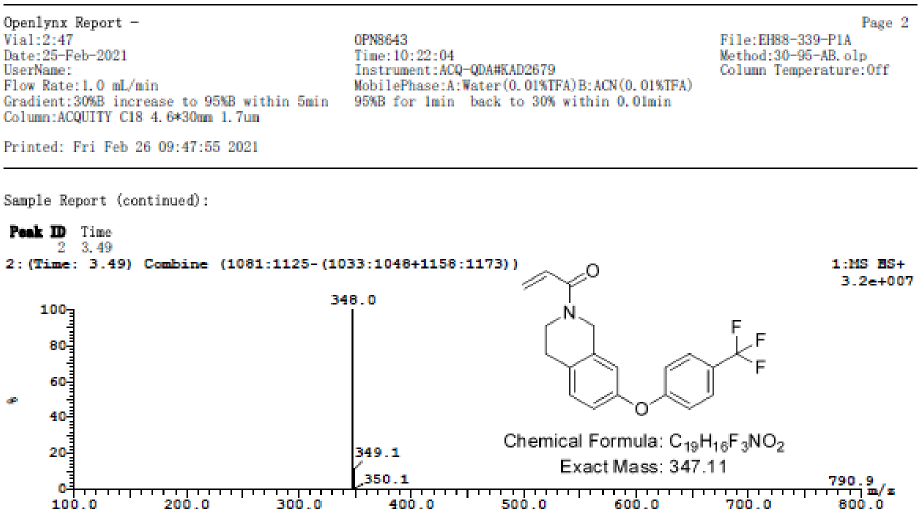

LCMS traces of OPN9643

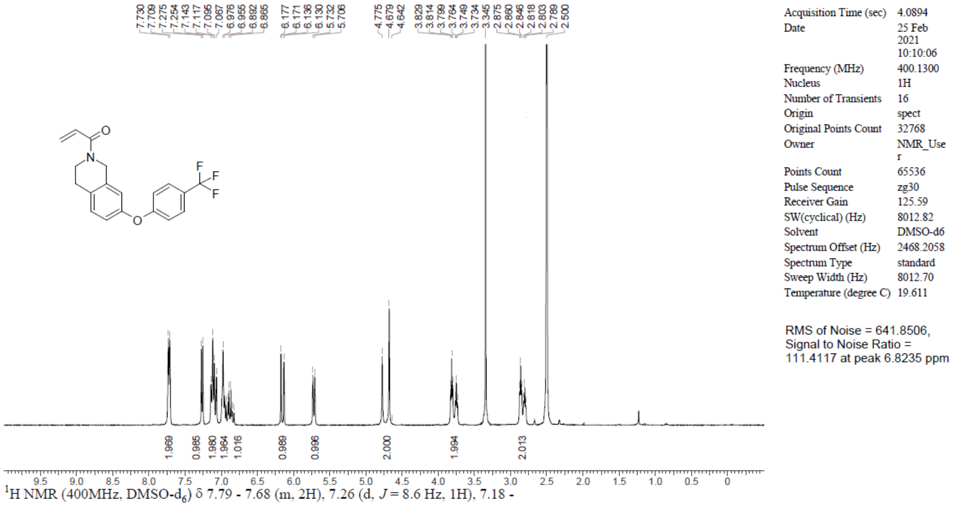

^{1}H NMR (400MHz, DMSO-d_6) δ 7.79 - 7.68 (m, 2H), 7.26 (d, J = 8.6 Hz, 1H), 7.18 - 7.09 (m, 2H), 7.08 - 6.94 (m, 2H), 6.94 - 6.79 (m, 1H), 6.15 (dd, J = 2.3, 16.6 Hz, 1H), 5.78 - 5.65 (m, 1H), 4.82 - 4.62 (m, 2H), 3.88 - 3.69 (m, 2H), 2.93 - 2.76 (m, 2H)

^{1}H-NMR spectrum of OPN9643

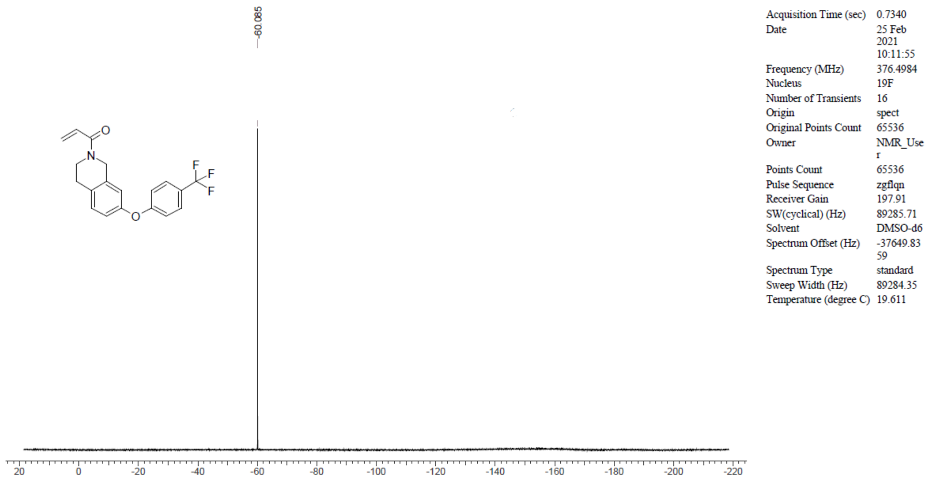

^{19}F-NMR spectrum of OPN9643

Openlynx Report -   Page 1
Vial:2:48
Date:22-Feb-2021
UserName:
Flow Rate:1.0 mL/min
Gradient:50%B increase to 95%B within 5min
Column:ACQUITY C18 4.6*30mm 1.7um

OPN9652
Time:16:51:45
Instrument:ACQ-QDA#KAD2679
MobilePhase:A:Water(0.01%TFA)B:ACN(0.01%TFA)
95%B for 1min back to 50% within 0.01min

File:EH98-266-P1B
Method:50-95-AB.olp
Column Temperature:Off

Printed: Tue Feb 23 10:26:08 2021

Sample Report:

Vial 2:48 File EH98-266-P1B Date 22-Feb-2021 Time 16:51:45 Method 50-95-AB.olp

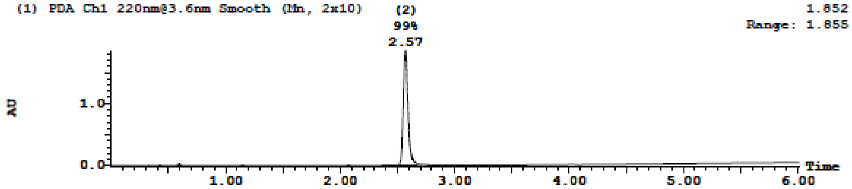

Peak Number	Time	AreaAbs	Area %Total	Width	Height
1	0.59	5.19e+002	0.57	0.05	2.31e+004
2	2.57	9.13e+004	99.43	0.27	1.85e+006

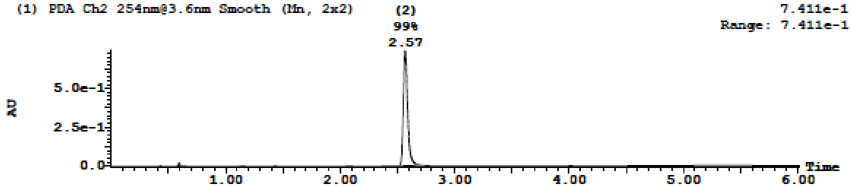

Peak Number	Time	AreaAbs	Area %Total	Width	Height
1	0.59	2.88e+002	0.86	0.04	1.89e+004
2	2.57	3.31e+004	99.14	0.21	7.39e+005

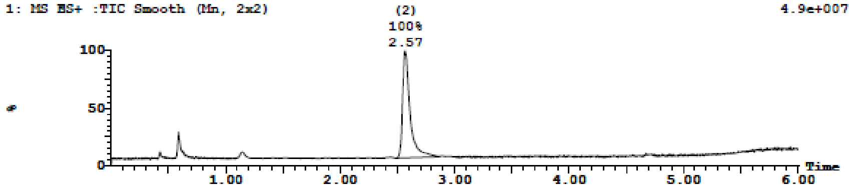

Peak Number	Time	AreaAbs	Area %Total	Width	Height
2	2.57	3.49e+006	100.00	0.37	4.51e+007

Openlynx Report -   Page 2
Vial:2:48
Date:22-Feb-2021
UserName:
Flow Rate:1.0 mL/min
Gradient:50%B increase to 95%B within 5min
Column:ACQUITY C18 4.6*30mm 1.7um

OPN9652
Time:16:51:45
Instrument:ACQ-QDA#KAD2679
MobilePhase:A:Water(0.01%TFA)B:ACN(0.01%TFA)
95%B for 1min back to 50% within 0.01min

File:EH98-266-P1B
Method:50-95-AB.olp
Column Temperature:Off

Printed: Tue Feb 23 10:26:08 2021

Sample Report (continued):

Peak ID	Time
2	2.57

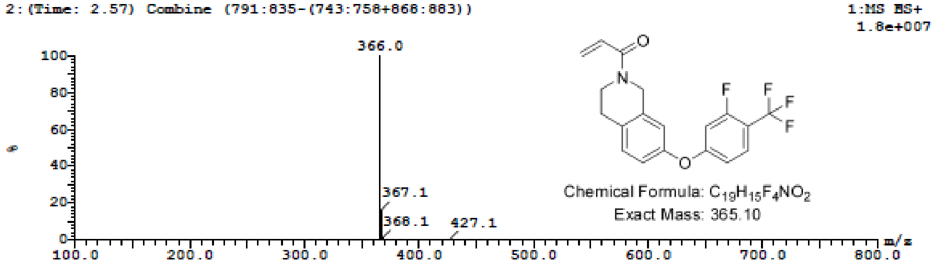

LCMS traces of OPN9652

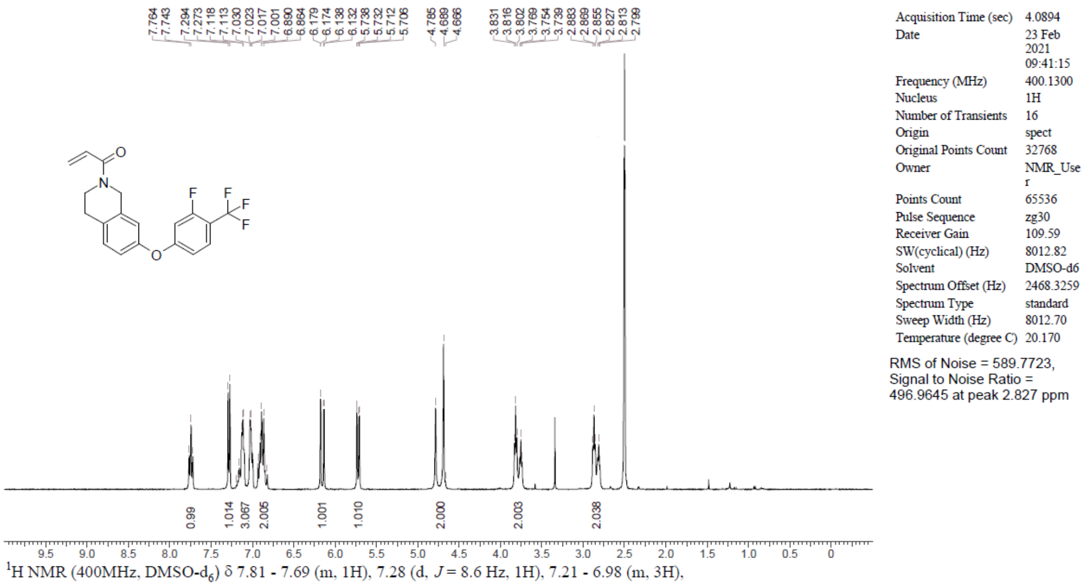

^{1}H NMR (400MHz, DMSO-d_6) δ 7.81 - 7.69 (m, 1H), 7.28 (d, J = 8.6 Hz, 1H), 7.21 - 6.98 (m, 3H), 6.95 - 6.78 (m, 2H), 6.16 (dd, J = 2.3, 16.6 Hz, 1H), 5.72 (dd, J = 2.3, 10.5 Hz, 1H), 4.81 - 4.65 (m, 2H), 3.86 - 3.72 (m, 2H), 2.93 - 2.76 (m, 2H)

^{1}H-NMR spectrum of OPN9652

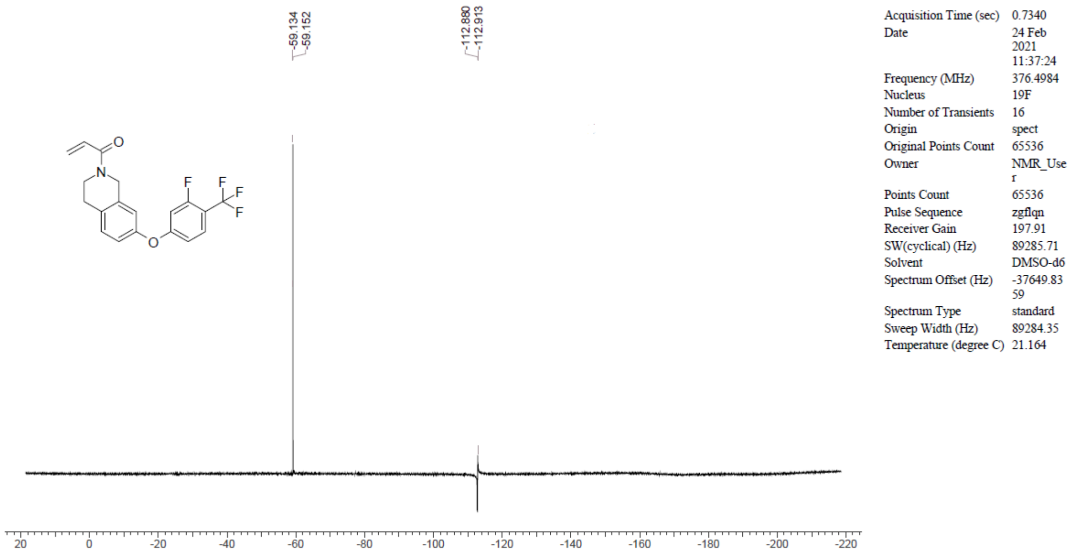

^{19}F-NMR spectrum of OPN9652

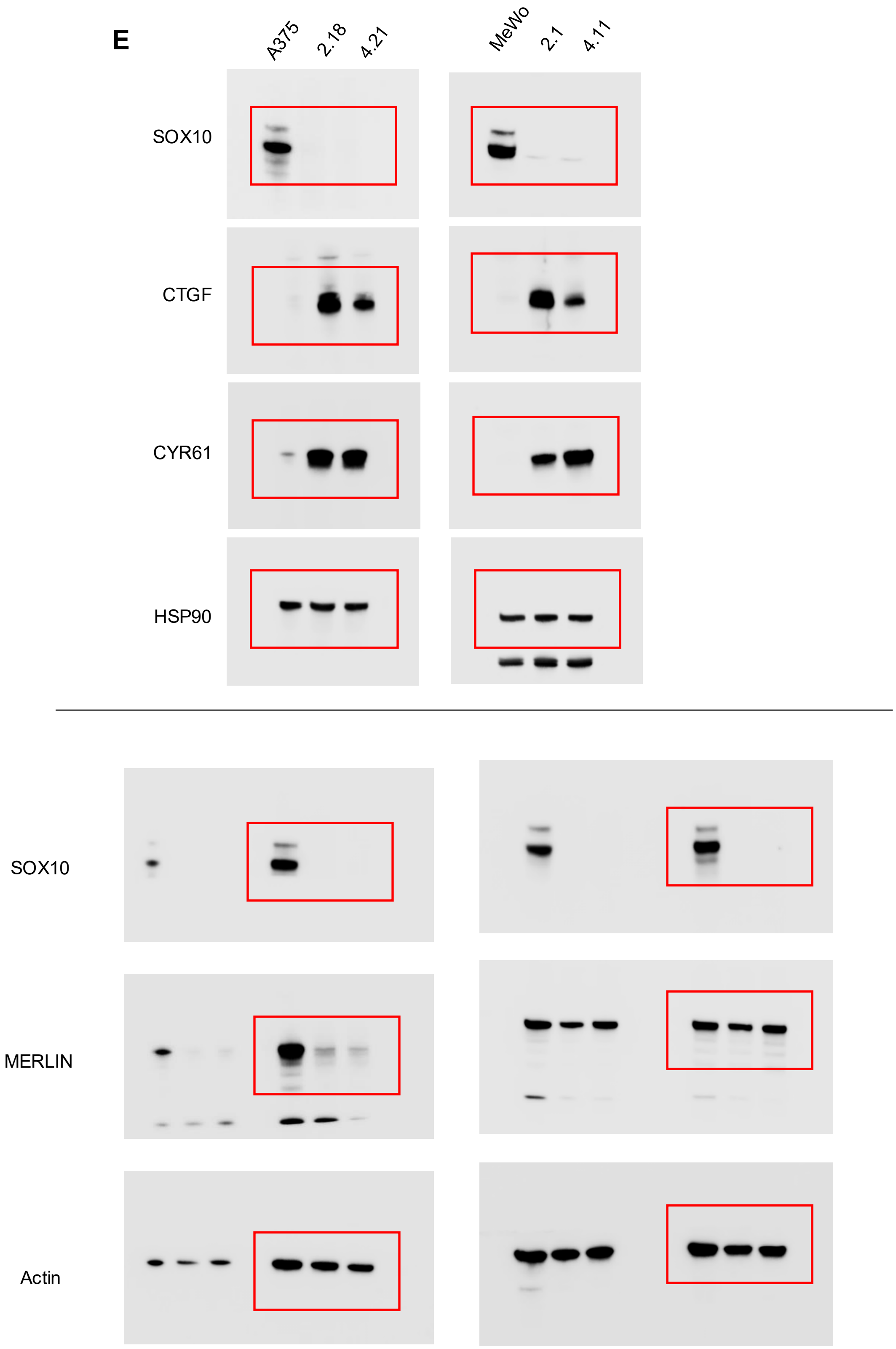
E
A375
2.18
4.21
MeWo
2.1
4.11
SOX10
CTGF
CYR61
HSP90
SOX10
MERLIN
Actin

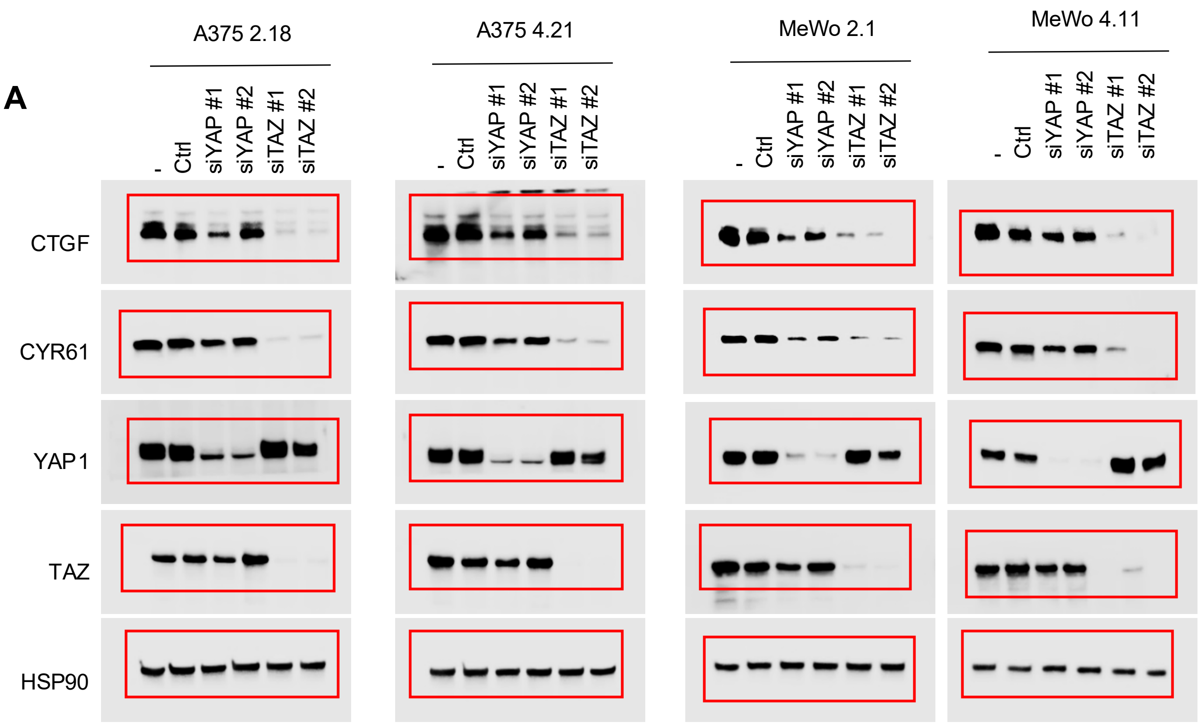
A
A375 2.18
A375 4.21
MeWo 2.1
MeWo 4.11
-
Ctrl
siYAP #1
siYAP #2
siTAZ #1
siTAZ #2
CTGF
CYR61
YAP1
TAZ
HSP90

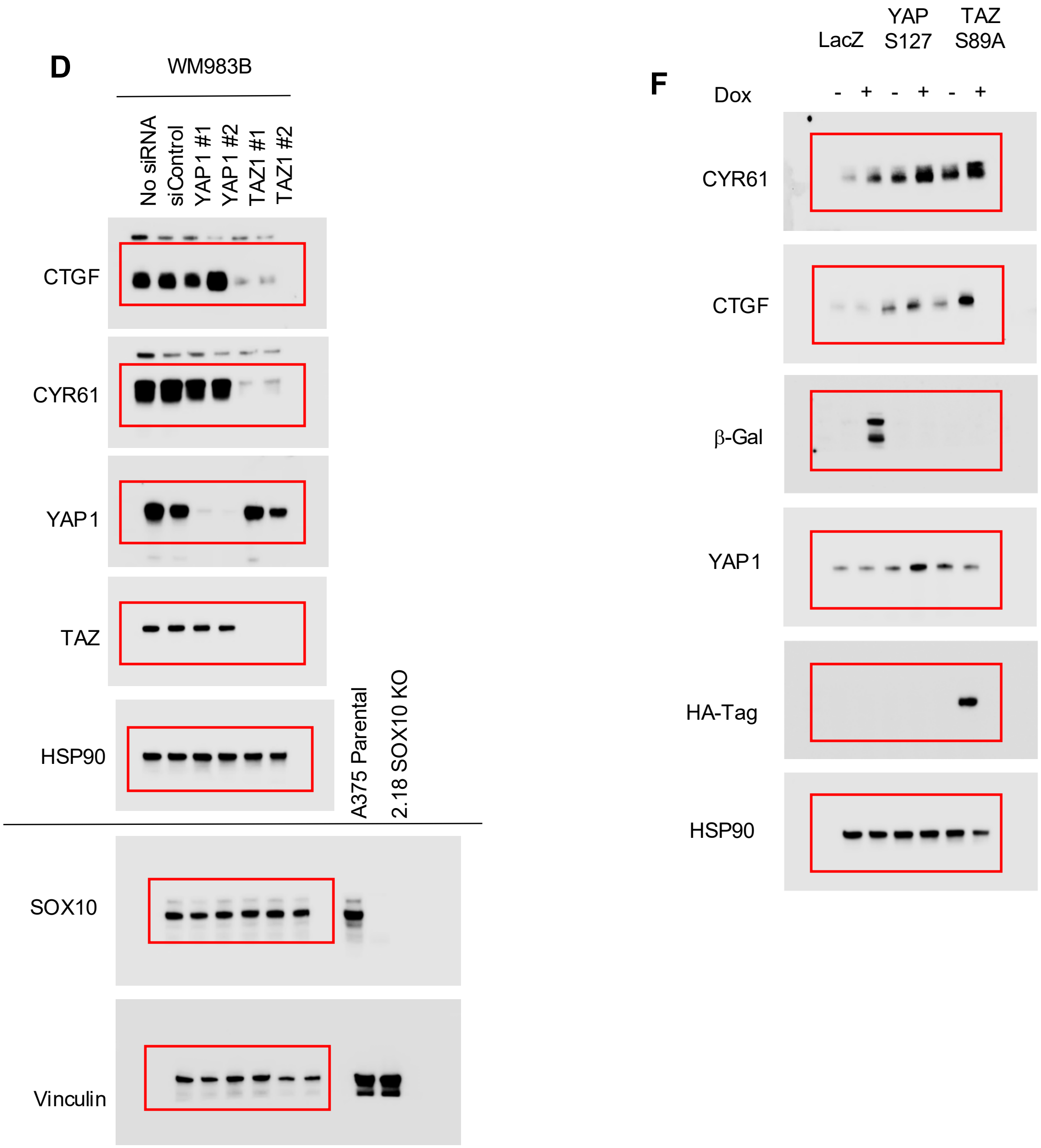
D
WM983B
No siRNA
siControl
YAP1 #1
YAP1 #2
TAZ1 #1
TAZ1 #2
CTGF
CYR61
YAP1
TAZ
HSP90
A375 Parental
2.18 SOX10 KO
SOX10
Vinculin
F
LacZ
YAP S127
TAZ S89A
Dox
- + - + - +
CYR61
CTGF
β-Gal
YAP1
HA-Tag
HSP90

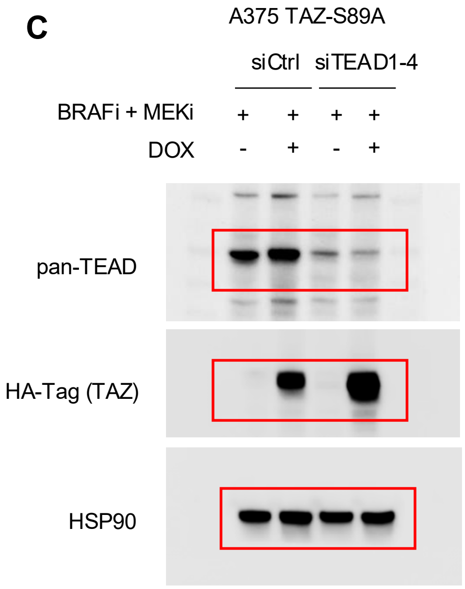
C
A375 TAZ-S89A
siCtrl
siTEAD1-4
BRAFi + MEKi
DOX
pan-TEAD
HA-Tag (TAZ)
HSP90

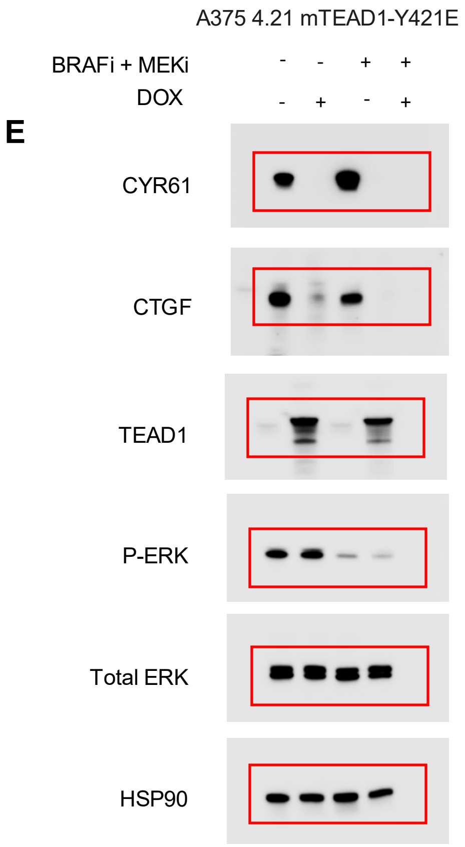
E
A375 4.21 mTEAD1-Y421E
BRAFi + MEKi
DOX
CYR61
CTGF
TEAD1
P-ERK
Total ERK
HSP90

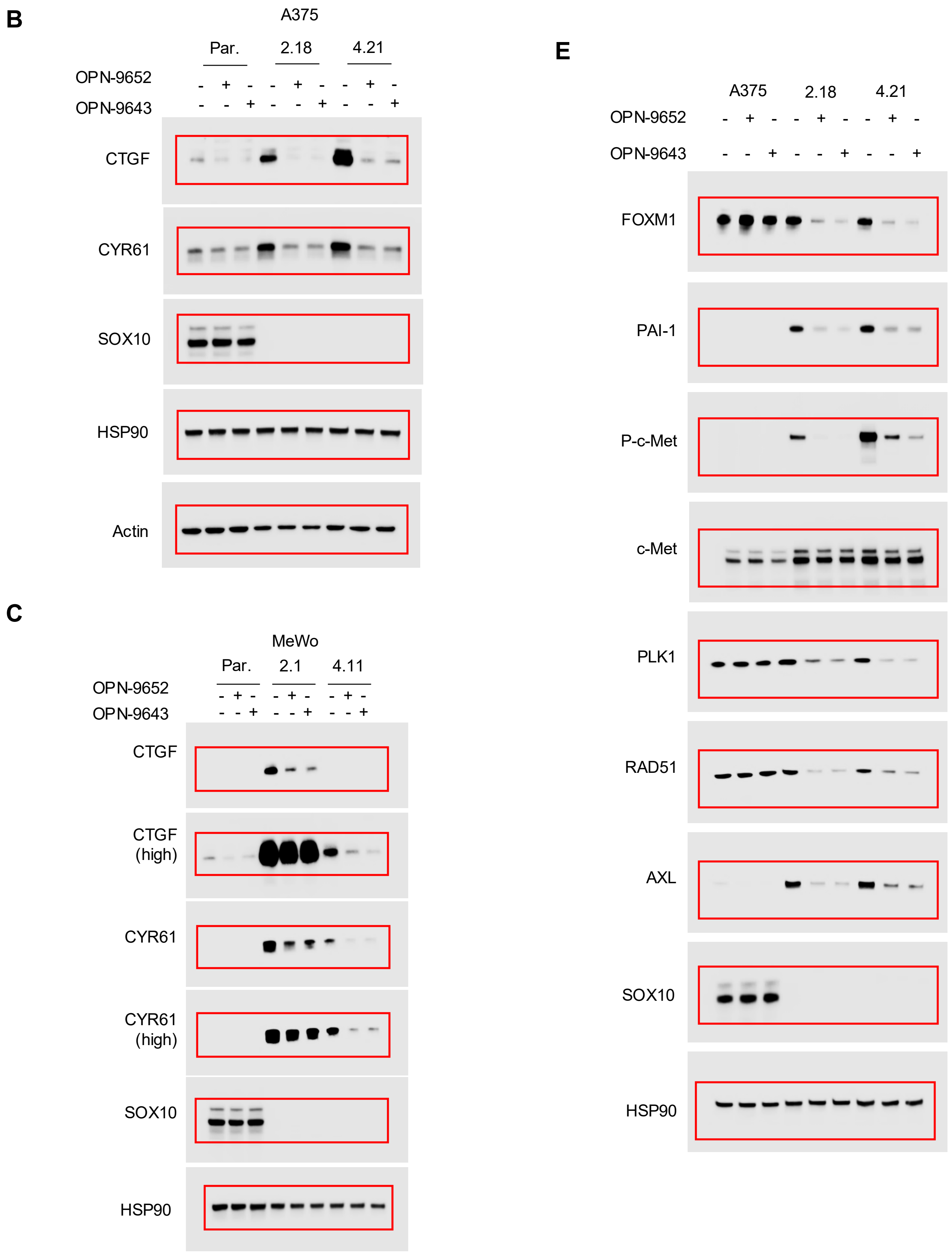
B
A375
Par.
2.18
4.21
OPN-9652
OPN-9643
CTGF
CYR61
SOX10
HSP90
Actin
C
MeWo
Par.
2.1
4.11
OPN-9652
OPN-9643
CTGF
CTGF (high)
CYR61
CYR61 (high)
SOX10
HSP90
E
A375
2.18
4.21
OPN-9652
OPN-9643
FOXM1
PAI-1
P-c-Met
c-Met
PLK1
RAD51
AXL
SOX10
HSP90

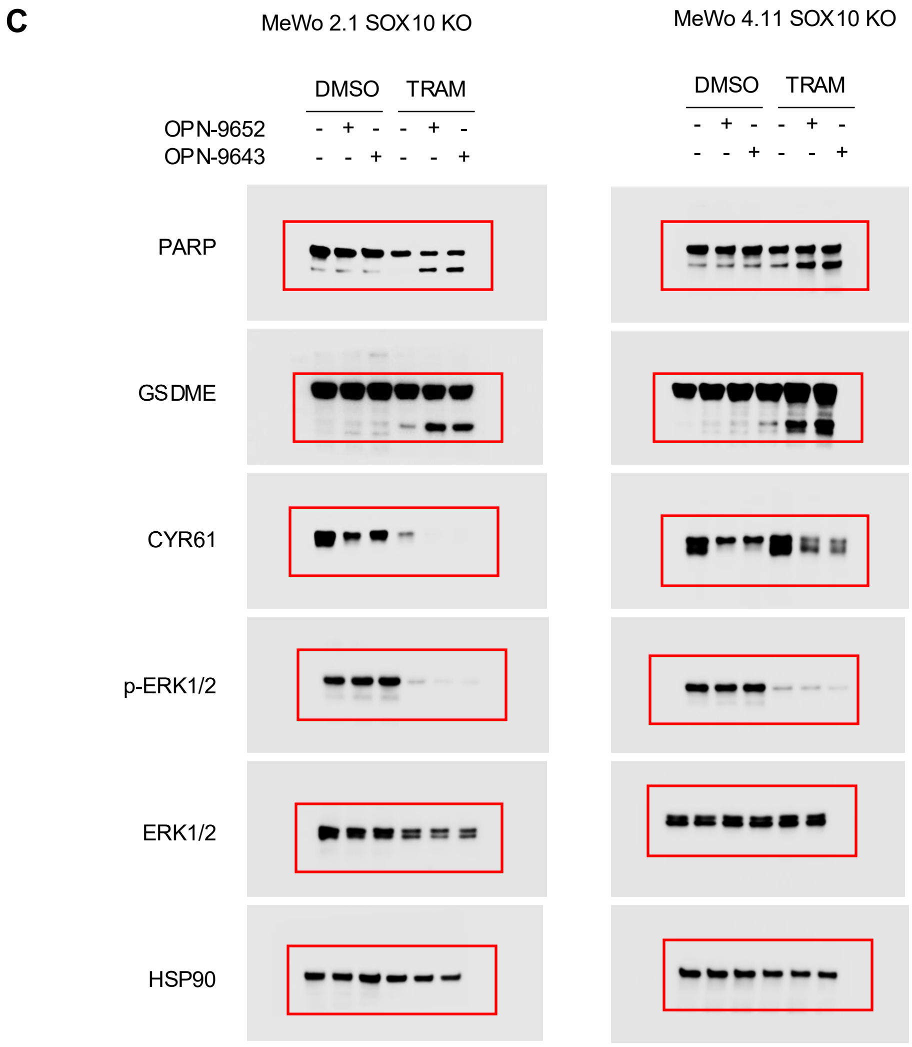
C
MeWo 2.1 SOX10 KO
MeWo 4.11 SOX10 KO
DMSO
TRAM
DMSO
TRAM
OPN-9652
OPN-9643
PARP
GSDME
CYR61
p-ERK1/2
ERK1/2
HSP90

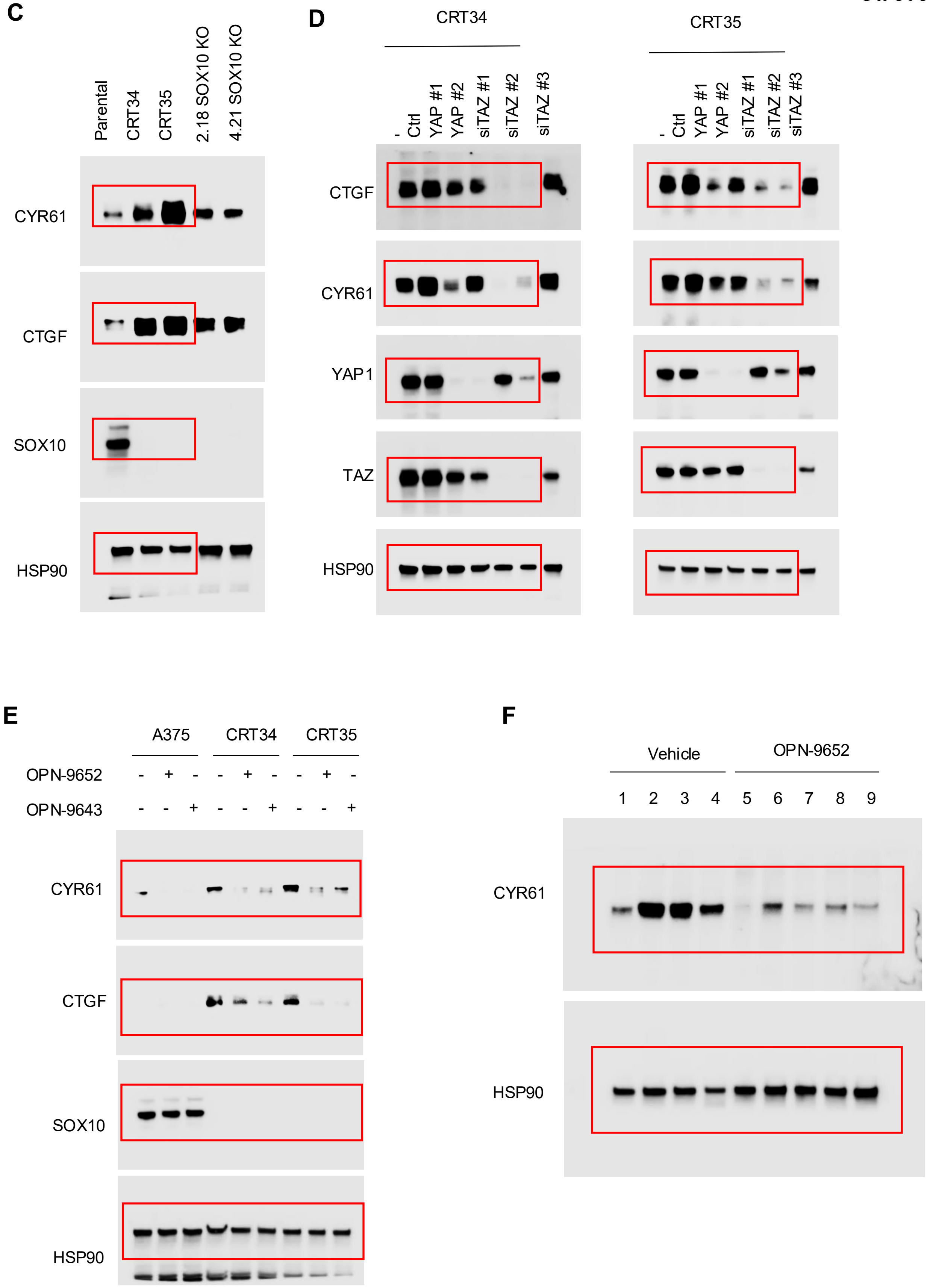
C
Parental
CRT34
CRT35
2.18 SOX10 KO
4.21 SOX10 KO
CYR61
CTGF
SOX10
HSP90
D
CRT34
CRT35
-
Ctrl
YAP #1
YAP #2
siTAZ #1
siTAZ #2
siTAZ #3
CTGF
CYR61
YAP1
TAZ
HSP90
E
A375
CRT34
CRT35
OPN-9652
OPN-9643
CYR61
CTGF
SOX10
HSP90
F
Vehicle
OPN-9652
1 2 3 4 5 6 7 8 9
CYR61
HSP90

Ott et al., Supplementary Figure 1

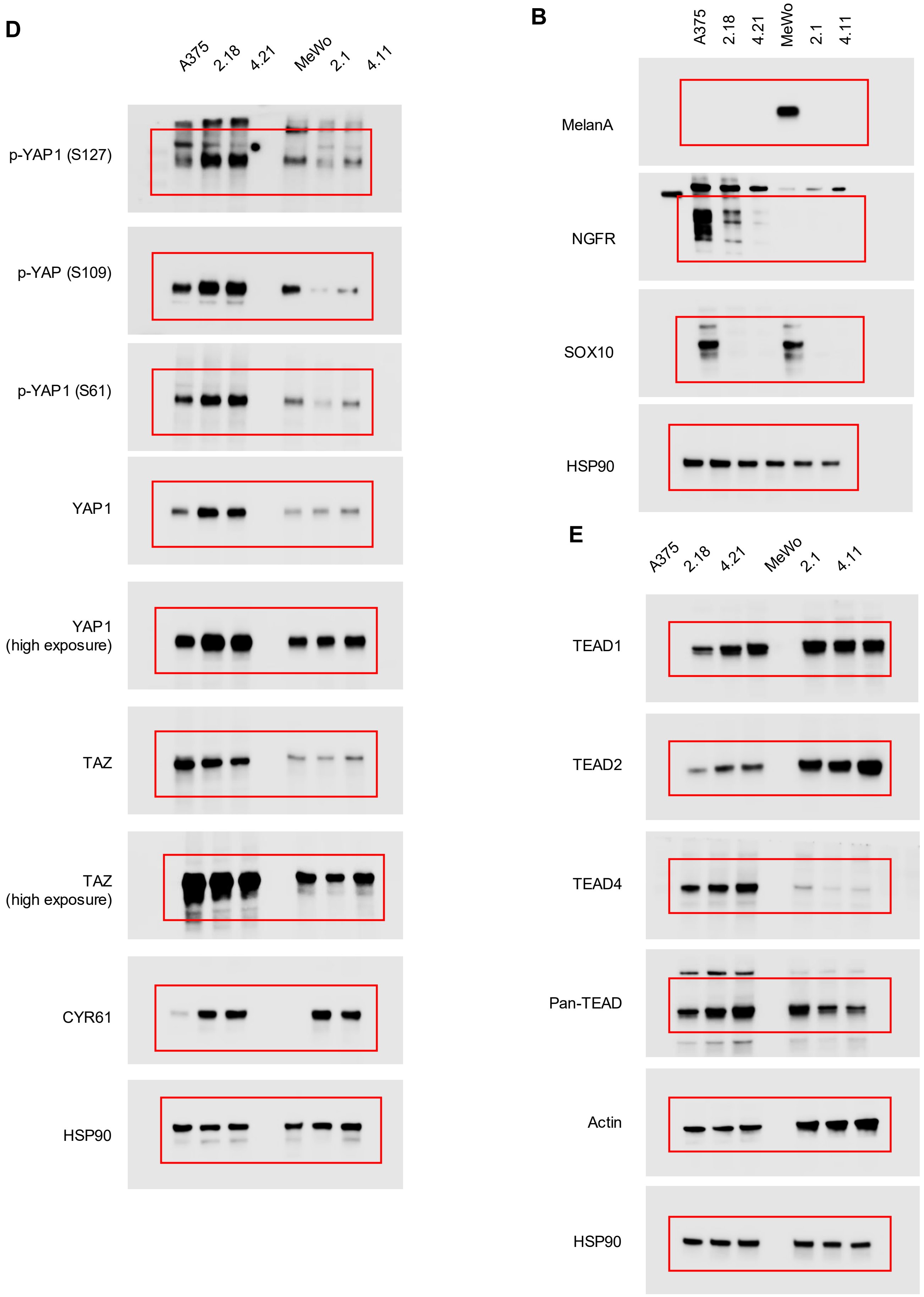

E

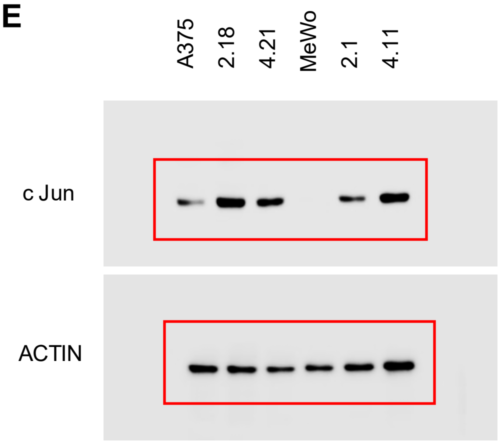
A375
2.18
4.21
MeWo
2.1
4.11
c Jun
ACTIN

F

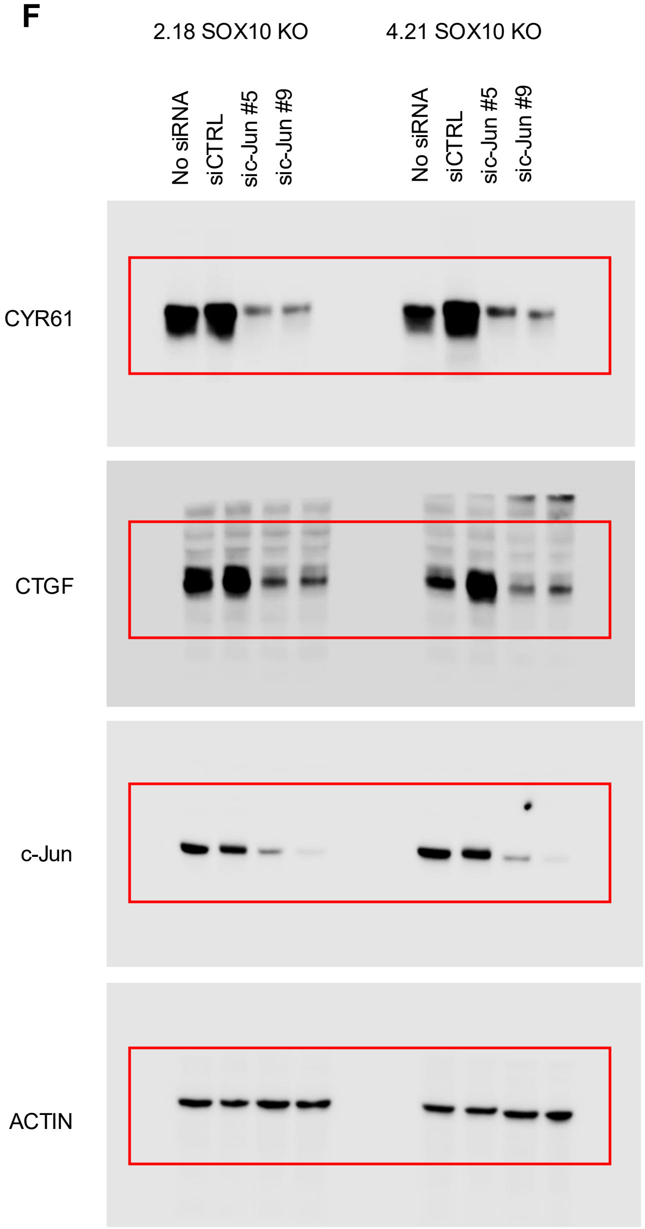
2.18 SOX10 KO
4.21 SOX10 KO
No siRNA
siCTRL
sic-Jun #5
sic-Jun #9
No siRNA
siCTRL
sic-Jun #5
sic-Jun #9
CYR61
CTGF
c-Jun
ACTIN

B

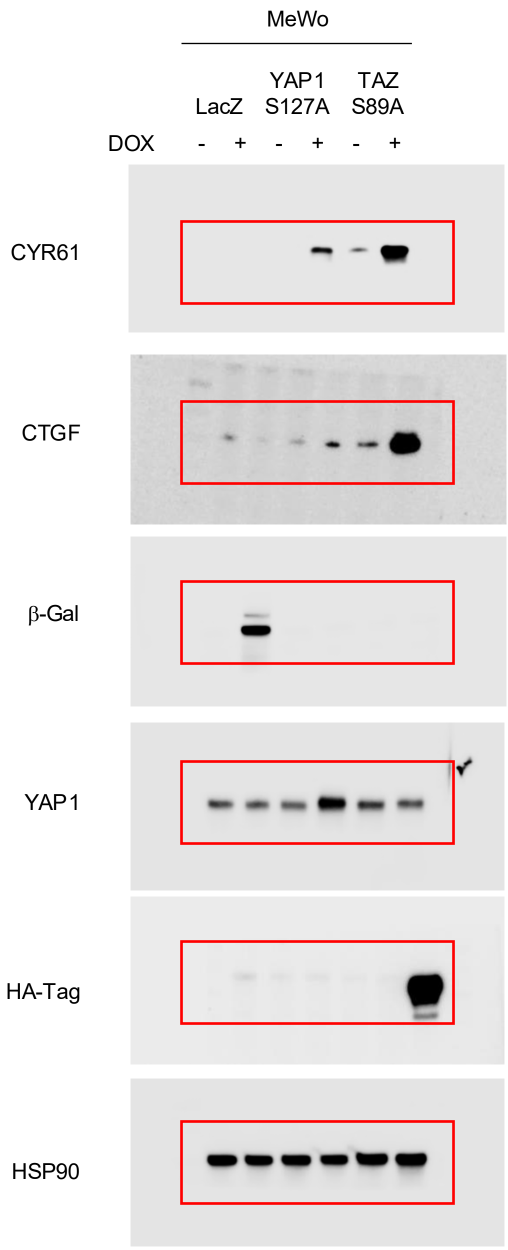
MeWo
LacZ
YAP1 S127A
TAZ S89A
DOX
- + - + - +
CYR61
CTGF
β-Gal
YAP1
HA-Tag
HSP90

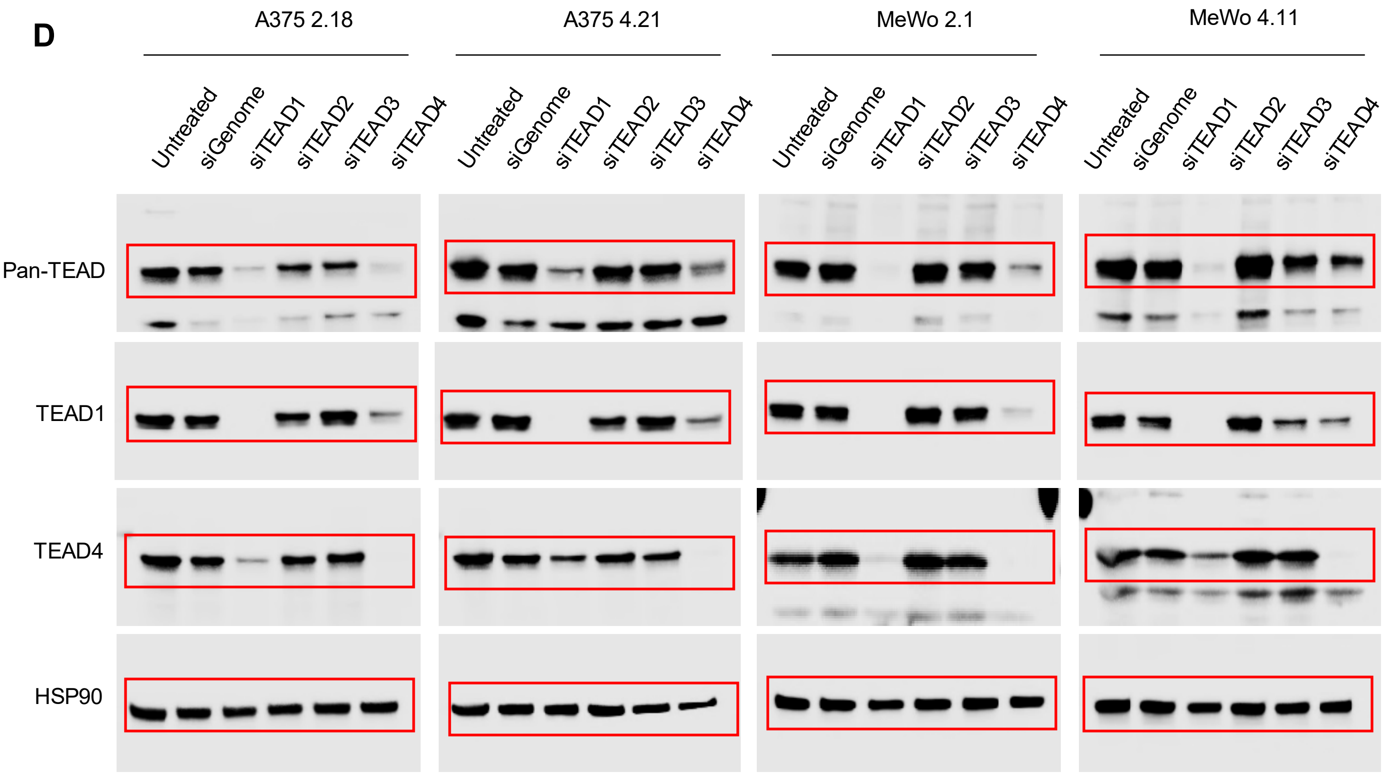
D
A375 2.18
A375 4.21
MeWo 2.1
MeWo 4.11
Untreated
siGenome
siTEAD1
siTEAD2
siTEAD3
siTEAD4
Pan-TEAD
TEAD1
TEAD4
HSP90

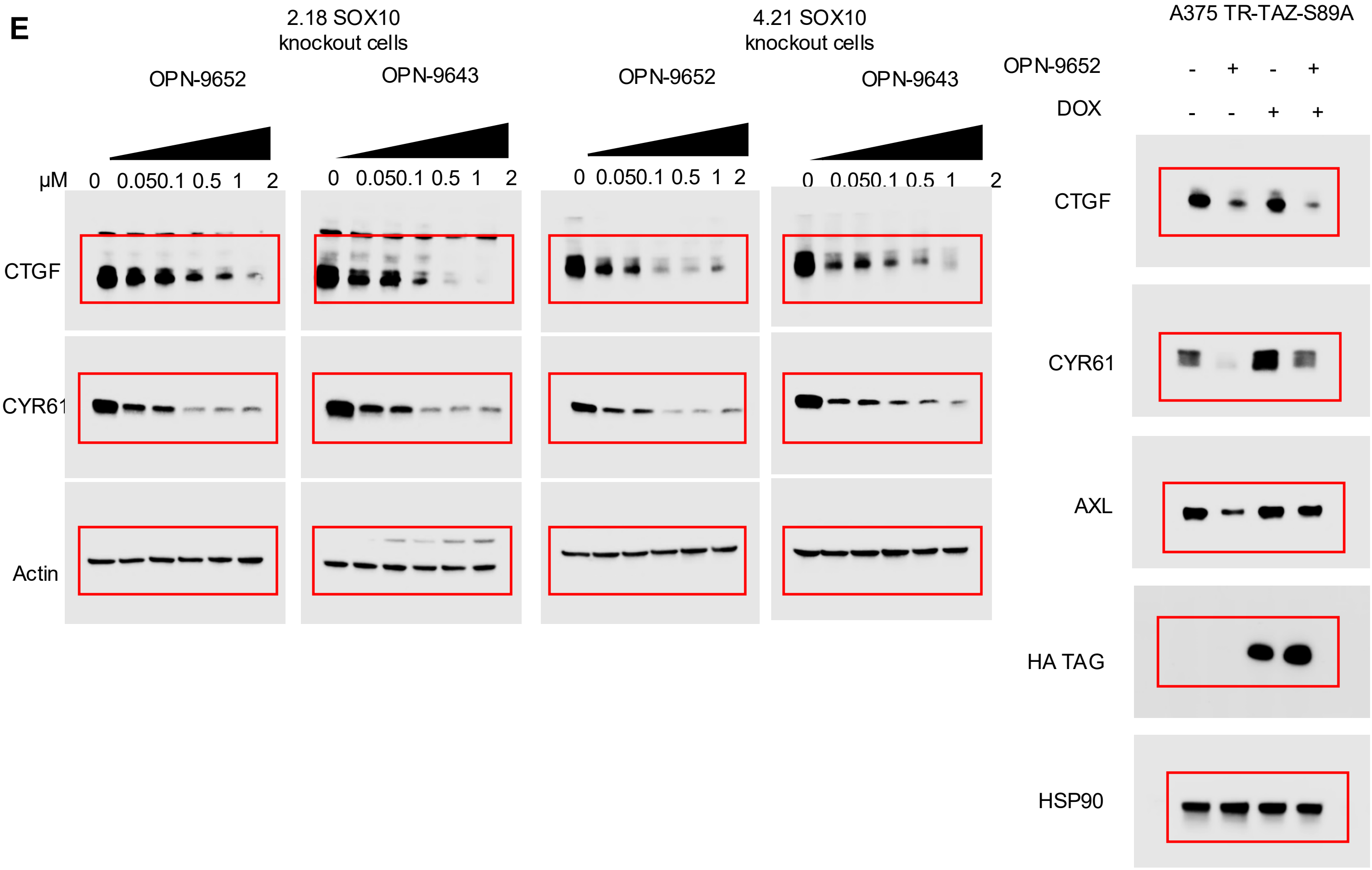
E
2.18 SOX10 knockout cells
4.21 SOX10 knockout cells
OPN-9652
OPN-9643
μM 0 0.05 0.1 0.5 1 2
CTGF
CYR61
Actin
A375 TR-TAZ-S89A
OPN-9652 - + - +
DOX - - + +
AXL
HA TAG
HSP90

B

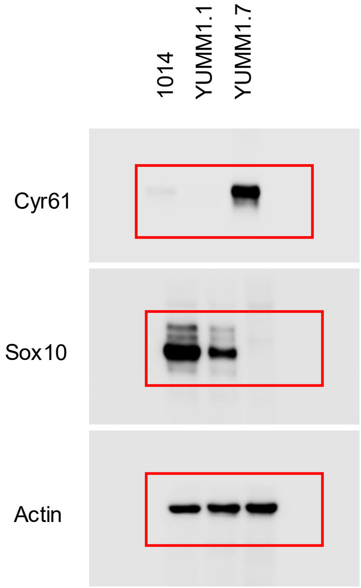

Supplementary References

1. Rambow, F., et al., *Toward minimal residual disease-directed therapy in melanoma.* Cell, 2018. **174**(4): p. 843-855 e19.
2. Zhang, X., et al., *The Hippo pathway oncoprotein YAP promotes melanoma cell invasion and spontaneous metastasis.* Oncogene, 2020. **39**(30): p. 5267-5281.
3. Verfaillie, A., et al., *Decoding the regulatory landscape of melanoma reveals TEADS as regulators of the invasive cell state.* Nat Commun, 2015. **6**: p. 6683.
