# Supplementary material for: Targeting TAZ-TEAD in minimal residual disease enhances the duration of targeted therapy in melanoma models
Source: Nat Commun. 2025 Nov 5;16:9655. doi: 10.1038/s41467-025-64682-7 (PMC12589586; doi:10.1038/s41467-025-64682-7)
Supplement: Supplementary file 2 — Description of Additional Supplementary Files [file 41467_2025_64682_MOESM2_ESM.pdf]

Title: Supplementary Data 1

Description: Targeting TAZ-TEAD in minimal residual disease enhances the duration of targeted therapy in melanoma models
