# Supplementary material for: Targeting TAZ-TEAD in minimal residual disease enhances the duration of targeted therapy in melanoma models
Source: Nat Commun. 2025 Nov 5;16:9655. doi: 10.1038/s41467-025-64682-7 (PMC12589586; doi:10.1038/s41467-025-64682-7)
Supplement: Supplementary file 4 — Reporting Summary [file 41467_2025_64682_MOESM4_ESM.pdf]

nature portfolio

Corresponding author(s): Andrew E. Aplin

Last updated by author(s): Jul 29, 2025

Reporting Summary

Nature Portfolio wishes to improve the reproducibility of the work that we publish. This form provides structure for consistency and transparency in reporting. For further information on Nature Portfolio policies, see our Editorial Policies and the Editorial Policy Checklist.

Statistics

For all statistical analyses, confirm that the following items are present in the figure legend, table legend, main text, or Methods section.

n/a	Confirmed	
☐	☒	The exact sample size (*n*) for each experimental group/condition, given as a discrete number and unit of measurement
☐	☒	A statement on whether measurements were taken from distinct samples or whether the same sample was measured repeatedly
☐	☒	The statistical test(s) used AND whether they are one- or two-sided<br>*Only common tests should be described solely by name; describe more complex techniques in the Methods section.*
☐	☒	A description of all covariates tested
☐	☒	A description of any assumptions or corrections, such as tests of normality and adjustment for multiple comparisons
☐	☒	A full description of the statistical parameters including central tendency (e.g. means) or other basic estimates (e.g. regression coefficient) AND variation (e.g. standard deviation) or associated estimates of uncertainty (e.g. confidence intervals)
☐	☒	For null hypothesis testing, the test statistic (e.g. *F*, *t*, *r*) with confidence intervals, effect sizes, degrees of freedom and *P* value noted<br>*Give P values as exact values whenever suitable.*
☒	☐	For Bayesian analysis, information on the choice of priors and Markov chain Monte Carlo settings
☐	☒	For hierarchical and complex designs, identification of the appropriate level for tests and full reporting of outcomes
☐	☒	Estimates of effect sizes (e.g. Cohen's *d*, Pearson's *r*), indicating how they were calculated

Our web collection on statistics for biologists contains articles on many of the points above.

Software and code

Policy information about availability of computer code

Data collection	The data generated for this paper consist of high-throughput RNA sequencing data obtained via Illumina instruments as FASTQ files.<br><br>Raw reads from publicly-available SRA RNA-seq datasets were downloaded using SRA toolkit (v 2.10.4).<br><br>scRNA-seq data collected from GEO were downloaded from GEO accession web pages.<br><br>DepMap CRISPR screen data were downloaded from DepMap's custom download page (https://depmap.org/portal/download/custom/)
Data analysis	- RNA-Seq datasets: STAR (v2.7.10a); RSEM (v1.3.3); DESeq2 (v1.28.1) ; fGSEA (v1.24.0); R-Project (v 4.2.2, 4.3.2); GSEA (4.2.3)<br><br>- scRNA-Seq datasets: Seurat (v4.3.0) ; R-Project (v4.3.2)<br><br>- visualization: VennDiagram (v 1.6.20 https://CRAN.R-project.org/package=VennDiagram); ggplot2 (v 3.3.2 https://ggplot2.tidyverse.org/); pheatmap (v 1.0.12 https://CRAN.R-project.org/package=pheatmap)

For manuscripts utilizing custom algorithms or software that are central to the research but not yet described in published literature, software must be made available to editors and reviewers. We strongly encourage code deposition in a community repository (e.g. GitHub). See the Nature Portfolio guidelines for submitting code & software for further information.

Data

Policy information about availability of data

All manuscripts must include a data availability statement. This statement should provide the following information, where applicable:

- Accession codes, unique identifiers, or web links for publicly available datasets
- A description of any restrictions on data availability
- For clinical datasets or third party data, please ensure that the statement adheres to our policy

Co-crystal structure of TEAD1 with OPN-9652 has been submitted to the worldwide protein data bank (wwPDB), under accession code: PDB ID 8S6Y

YAP1/TAZ knockdown RNA Seq datasets associated with this publication can be found under GEO accession numbers: GSE259388

OPN-9652 and OPN-9643 treated RNA Seq datasets associated with this publication can be found under GEO accession numbers: GSE259389

SRP306463: MeWo parental and CRISPR SOX10 bulk RNA-seq samples
SRP329298: A375 parental and CRISPR SOX10 bulk RNA-seq samples
SRP329298: A375 parental and BRAFi+MEKi-resistant (CRT) bulk RNA-seq samples
SRP329297: 1205LuTR parental and BRAFi-resistant (PBRT) bulk RNA-seq samples
SRP029434: A375 parental and A375 shSOX10 bulk RNA-seq samples

GSE116237: Rambow scRNA-seq data

DepMap: CRISPR screen Chronos scores (v22Q2)

Fig 1: SRP306463, SRP329298, SRP029434 and GSE116237
Fig 2: GSE259388
Fig 3: DepMap
Fig 6:GSE259389
Fig 8: SRP329298 and SRP329297

Research involving human participants, their data, or biological material

Policy information about studies with human participants or human data. See also policy information about sex, gender (identity/presentation), and sexual orientation and race, ethnicity and racism.

Reporting on sex and gender	N/A
Reporting on race, ethnicity, or other socially relevant groupings	N/A
Population characteristics	N/A
Recruitment	N/A
Ethics oversight	N/A

Note that full information on the approval of the study protocol must also be provided in the manuscript.

Field-specific reporting

Please select the one below that is the best fit for your research. If you are not sure, read the appropriate sections before making your selection.

☒ Life sciences ☐ Behavioural & social sciences ☐ Ecological, evolutionary & environmental sciences

For a reference copy of the document with all sections, see nature.com/documents/nr-reporting-summary-flat.pdf

Life sciences study design

All studies must disclose on these points even when the disclosure is negative.

Sample size	For mouse experiments, the sample size was established to provide 81% power to detect differences among 2 means, corresponding to the effect sizes of 0.5, 1.0, and 1.5 for comparison of the treated group vs. vehicle control, assuming a common standard deviation and using an F test with alpha=0.025.
Data exclusions	Mice that were found dead due to unexplained circumstances were censored from the study.
Replication	For in vitro assays associated with statistical analysis: Cell growth assay (IncuCyte) and 3D spheroid, were performed as 3 independent

	experiments (biological replicates) for rigor and to perform statistical analysis.
Randomization	Mice harboring tumors with similar sizes were randomly divided into 2 different cohorts.
Blinding	N/A

Reporting for specific materials, systems and methods

We require information from authors about some types of materials, experimental systems and methods used in many studies. Here, indicate whether each material, system or method listed is relevant to your study. If you are not sure if a list item applies to your research, read the appropriate section before selecting a response.

Materials & experimental systems

n/a	Involved in the study
☐	☒ Antibodies
☐	☒ Eukaryotic cell lines
☒	☐ Palaeontology and archaeology
☐	☒ Animals and other organisms
☒	☐ Clinical data
☒	☐ Dual use research of concern
☒	☐ Plants

Methods

n/a	Involved in the study
☒	☐ ChIP-seq
☒	☐ Flow cytometry
☒	☐ MRI-based neuroimaging

Antibodies

Antibodies used	Primary antibodies SOX10 (#89356, 1:1000) CTGF (#86641, 1:1000), CYR61 (#14479S, 1:1000), HSP90 (#4877, 1:3000), Merlin (#12888, 1:1000), TAZ (#70148S, 1:1000), HA-Tag (#2367, 1:1000), TEAD1 (#12292S, 1:1000), P-ERK (Thr202/Tyr204) (#9101, 1:1000), Total ERK (#9102, 1:1000) pan-TEAD (#13295S, 1:1000), FOXM1 (#5436, 1:1000), PAI-1 (#49536, 1:1000), and P-c-Met (Tyr1234/1235) (#3077, 1:1000), and c-Met (#8198, 1:1000), PLK1 (#4513S, 1:1000), RAD51 (#8875, 1:1000), and AXL (#8661, 1:1000) were purchased from Cell Signaling Technology.-Gal (#Z378A, 1:1000) was purchased from Promega. YAP1 (#ab52771, 1:1000), TEAD4 (#ab58310, 1:1000) antibodies were purchased from Abcam. Actin (#A2066, 1:2000) antibodies were purchased from Santa Cruz Biotechnology. Secondary antibodies Goat Anti-Mouse IgG (#401215, 1:4000) and Goat Anti-Rabbit IgG (#401315, 1:4000) were purchased from Sigma-Aldrich Co.
Validation	Primary antibodies were validated in preliminary experiments with cell pellets expressing the target and the appropriate knockdown control to verify antibody specificity.

Eukaryotic cell lines

Policy information about cell lines and Sex and Gender in Research

Cell line source(s)	MeWo cells were kindly donated by Dr. Barbara Bedogni, when at Case Western Reserve, Cleveland, OH in 2014, A375 parental cells were purchased from ATCC in 2005, and WM983B were provided by Dr. Meenhard Herlyn, The Wistar Institute, Philadelphia, PA in 2005.
Authentication	Short-tandem repeat analysis was performed to authenticate cell lines. All cell lines matched known profiles.
Mycoplasma contamination	Cells were assayed for mycoplasma contamination every two months with MycoScope Kit (Genlantis).
Commonly misidentified lines (See ICLAC register)	None

Animals and other research organisms

Policy information about studies involving animals; ARRIVE guidelines recommended for reporting animal research, and Sex and Gender in Research

Laboratory animals	NOD.Cg-Prkdcscid Il2rgtm1Wjl/SzJ (NSG) mice male and female 6-8 weeks old.
Wild animals	The study did not involve wild animals
Reporting on sex	Male (8) and Female (14) mice were used in this study, but split as equally as possible between experimental arms.
Field-collected samples	All animals are provided with food and water ad libitum, and housed in cages (with a maximum of 5 mice/cage) in a temperature and humidity-controlled environment. Animals are maintained in housing conditions that allow for normal species behavior to minimize the development of abnormal behaviors, and have access to humane and veterinary care.<br>Mice were sacrificed when the tumor volume was greater than 1000 mm3.
Ethics oversight	Animal experiments were performed at a Thomas Jefferson University facility that is accredited by the Association for the Assessment

and Accreditation of Laboratory Animal Care. The Institutional Animal Care and Use Committee approved these studies.

Note that full information on the approval of the study protocol must also be provided in the manuscript.

Plants

Seed stocks	N/A
Novel plant genotypes	N/A
Authentication	N/A
